# First genetic evaluation of a wild population of *Crocodylus intermedius*: New insights for the recovery of a Critically Endangered species

**DOI:** 10.1371/journal.pone.0311412

**Published:** 2024-10-03

**Authors:** Nicolás Castillo-Rodríguez, Ana M. Saldarriaga-Gómez, Rafael Antelo, Mario Vargas-Ramírez

**Affiliations:** 1 Grupo de Biodiversidad y Conservación Genética, Instituto de Genética, Universidad Nacional de Colombia, Bogotá, Colombia; 2 Department of Biology, University of Kentucky, Lexington, Kentucky, United States of America; 3 Estación de Biología Tropical Roberto Franco, Universidad Nacional de Colombia, Villavicencio, Colombia; 4 Department of Biological Sciences, Fordham University, Bronx, New York, United Stated of America; 5 WWF-Bolivia, Santa Cruz, Bolivia; Senckenberg am Meer Deutsches Zentrum fur Marine Biodiversitatsforschung, GERMANY

## Abstract

During the second third of last century, the Orinoco Crocodile (*Crocodylus intermedius*) underwent a hunting process driven by the demand from the North American, European, and Japanese leather industry, resulting in a sharp decline of its populations. Currently, only two known remaining populations of this Critically Endangered species persist in the Colombian Orinoquía: in the Guayabero-Duda-Lozada and the Cravo Norte-Ele-Lipa River Systems. The latter has been the only population subject of study, including recent surveys and local conservation initiatives such as egg and hatchling ranching. Despite suggestions for population recovery based on the observed increase in clutches in the area, information regarding its genetic status has been pending assessment. This research aims to provide a genetic characterization of this remaining population and to evaluate the diversity recovered during a period of the egg ranching initiative. For this purpose, we utilized variable molecular markers, specifically 17 microsatellite loci, nuclear DNA. Despite revealing intermediate levels of genetic diversity, we identified an effective population size of 11.5–17, well below the minimum values proposed for short-term subsistence. While no evidence of inbreeding was found, it is acknowledged as a potential risk based on the population’s history. Additionally, we detected a historical bottleneck possibly influenced by arid periods affecting the region since the Pleistocene. While the evaluated population presents a unique opportunity for *C*. *intermedius* conservation, it also exposes a high risk of entering the extinction vortex. The primary action to be taken is to support the egg and hatchling ranching program, which successfully recovered most of the genetic diversity present in the population.

## Introduction

*Crocodylus intermedius*, commonly known as the Orinoco Crocodile, is one of the most endangered species in the Neotropics [1, 2]. Historically, this species thrived across the lowlands of the Orinoco River basin in Colombia and Venezuela, inhabiting diverse aquatic ecosystems, including rivers in tropical forests and residually piedmont streams in the foothills of the Andean mountains [3, 4]. However, from 1928 to 1960s, *C*. *intermedius* experienced a staggering population decline due to an unsustainable commercial hunting process driven by the demand from the North American, European, and Japanese leather industry [3]. This decline, coupled with habitat loss and the collection of eggs for local consumption, has resulted in the Orinoco Crocodile being classified as Critically Endangered on the IUCN Red List [5], and listed in Appendix I of CITES [6]. Currently, the presence of *C*. *intermedius* in the wild primarily involves isolated individuals, such as those found in the Vichada River [7] or small groups in the Arauca, Manacacías, Meta, and Yucao rivers [8]. Additionally, a few population relics have been identified as regional habitat priorities or crocodile conservation units, specifically the Cojedes System and certain localities in the Apure State in Venezuela, and the Duda-Guayabero-Lozada/Cravo Norte-Ele-Lipa River Systems in Colombia [2]. Among these remnants, the Cravo Norte-Ele-Lipa River Systems population stands out as the only one systematically studied in Colombia. Surveys conducted in 1994–1995, 2000–2001, 2012, and 2014–2015 [9–11] have provided valuable insights. A concurrent rise in observed nests has been noted, with reports ranging from seven to 11 nests between 1994 and 2012 [10–12]. Anzola and Antelo [13] reported 24 nests from December 2014 to April 2015, further suggesting a positive trend in population recovery [13]. Current initiatives include local efforts for crocodile conservation, such as egg [8] and hatchling ranching. Despite these positive actions, the genetic status of this population and the extent to which the egg and hatchling ranching initiatives contribute to its genetic diversity recovery remain unexplored.

The integration of genetic evaluations is a pivotal component in comprehensive management plans for threatened populations and species. It enables access to crucial population-level parameters, including diversity indexes, and provides evidence of events such as bottlenecks or inbreeding [14]. This approach allows for assessing population status and guiding decisions on necessary management actions with the overarching goal of increasing population size while preserving genetic diversity [14, 15]. The ultimate aim is to conserve the population’s evolutionary potential, ensuring its ability to adapt to a changing environment [16, 17]. This perspective has been recognized and endorsed by the National Program for the Conservation of the Orinoco Crocodile (PROCAIMAN; [18]), a Colombian governmental action established in 1998 to prevent the extinction of this species within the country. PROCAIMAN acknowledges the significance of incorporating genetic assessments of the species and populations into conservation efforts [18].

This research aimed to conduct the initial genetic characterization of an *in-situ* remnant population of *C*. *intermedius* by assessing individuals within the crocodile conservation unit of the Cravo Norte-Ele-Lipa Rivers System. To achieve this goal, we employed the analysis of variable molecular markers; 17 microsatellite loci nuclear DNA (nDNA), to evaluate the genetic diversity of the population and gain insights into its demographic history. Additionally, we sought to use the same set of microsatellite loci to assess the diversity recovered during a period of the hatchling ranching initiative, comparing it to the diversity identified in the population. This research aims to contribute to the conservation and surveillance efforts for this emblematic and endangered crocodile.

## Materials and methods

### Ethics statement

PROCAIMAN and the Interinstitutional Action Plan for the Orinoco Crocodile Conservation [8] assigned the Roberto Franco Tropical Biological Station (EBTRF) of the National University of Colombia (UNAL) as the scientific institution responsible for developing the genetic characterization of *Crocodylus intermedius* populations. This research is a component of this task. It has strictly adhered to all relevant methodologies and ethical standards for handling and sampling crocodiles as developed by the EBTRF and approved by the Ethics Committee of the Science Faculty at UNAL and the Ministry of Environment and Sustainable Development of Colombia. This study did not involve the use of anesthesia, euthanasia, or any form of animal sacrifice. Comprehensive efforts were made to ensure the well-being and appropriate treatment of the crocodiles throughout the study.

### Sample collection

**Fig 1:**
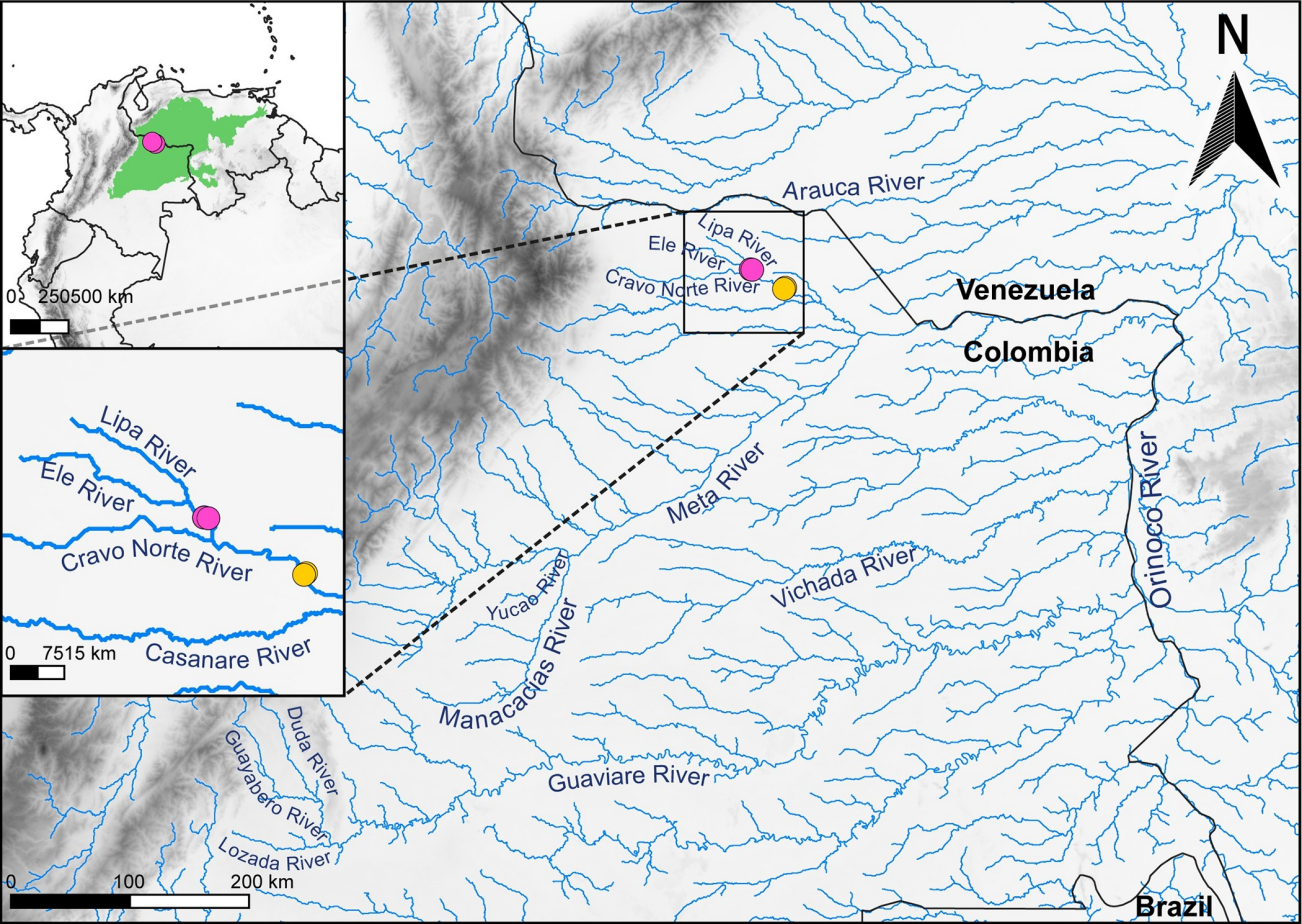
Sampling sites (depicted by yellow and pink dots) situated along the Cravo Norte and Ele rivers within the Arauca department, Colombia. The map was created with QGIS 3.14.1 [19]; boundaries were adapted from https://public.opendatasoft.com/explore/dataset/country_shapes/export/; elevation data from the Shuttle Radar Topography Mission (STRM) (https://www.earthdata.nasa.gov/sensors/srtm); hydrographic information from HydroRIVERS dataset [20]; and the historical distribution of *C*. *intermedius* is represented in green and was adapted from Balaguera-Reina and colleagues [5].

Two distinct datasets were employed in this investigation: the population dataset for conducting the genetic characterization of the *in-situ* population, and the conservation dataset for assessing the diversity recovered during a period of hatchling ranching. The population dataset comprised 38 caudal scale samples obtained between 2009 and 2017 from neonates or subadults individuals in the Cravo Norte (24 individuals) and Ele Rivers (14 individuals) within the Arauca department, Colombia (Fig 1 and S1 Table). Among these, 30 crocodiles were, at some stage of their life, part of the *ex-situ* breeding program coordinated by the EBTRF. Some were raised at the facility and later released back into their original location, while others are currently part of the breeding stock in the reproductive program.

For the conservation dataset, we exclusively included samples from 81 individuals out of 139 that were born through the hatchling ranching initiative in 2016 (S2 Table). The clutches were initially collected in the sector Playa Campo Abierto, which corresponds to a sand beach along the Cravo Norte River. All collected samples were preserved in 96% ethanol and stored at -20°C in the Colombian Biodiversity DNA and Tissue Bank (BTBC) at the Institute of Genetics (IGUN) of the UNAL.

### Laboratory procedures and genotyping

Genomic DNA from tissue was extracted using the NucleoSpin ® Tissue Kit (Machery-Nagel, Germany). A set of 17 microsatellites loci developed for the genus *Crocodylus* [21], *C*. *moreletii* [22], and *C*. *porosus* [23], previously employed for cross-amplification with *C*. *intermedius* by Rossi Lafferriere and colleagues [24] and Saldarriaga-Gómez and colleagues [25], was amplified (S3 Table). Polymerase Chain Reactions (PCR) and genotyping were conducted following the methods outlined in Castillo-Rodríguez and colleagues [26]. Laboratory procedures were executed in the Molecular Ecology Laboratory of the IGUN at the UNAL. Fragment length analysis was carried out by the Molecular Sequencing and Analysis Service (SSIGMOL)-IGUN-UNAL. The Gene-Mapper 3.7 (Applied Biosystems Foster City, CA) and Osiris 2.13.1 (NCBI) software were used for scoring fragment lengths.

### Data analysis

#### Population genetic variation

For the population data set, genotyping inconsistencies such as null allele frequencies at each locus and allele dropout were assessed with MICRO-CHECKER 2.2.3 [27]. GENEPOP 4.7.5 [28] was used to evaluate the tendency to Hardy Weinberg (HW) equilibrium for all loci using the implemented exact test, and genotypic linkage disequilibrium (LD) between each pair of loci using the log-likelihood ratio statistic. Significance levels were estimated using a Markov chain (MC) algorithm with 10,000 dememorization steps, 1,000 batches, and 10,000 iterations per batch. Bonferroni corrections were applied to both HW equilibrium and LD calculations. Observed allelic diversity (*A*_*Ob*_) and allelic richness (*A*_*R*_), were assessed with HP-RARE 1.0 [29], which integrates rarefaction to cope with the effects of sample size disparity between populations [30]. Observed (*H*_*O*_), and expected heterozygosities (*H*_*E*_) were assessed with ARLEQUIN 3.5.1.2 [31].

#### Inbreeding and effective population size

The inbreeding coefficient *F*_*IS*_ [32] was assessed in FSTAT 2.9.4 [30]. Its significance for excess or deficiency of heterozygotes [33] was evaluated in GENEPOP 4.7.5 [28] applying Bonferroni corrections.

Effective population size (*N*_*e*_) was assessed using two methods for comparative purposes. The first one corresponds to the sibship assignment method proposed by Wang [34] and implemented in COLONY [35]. It assumes that the smaller the population, the higher the probability that two randomly taken individuals are sibs; *N*_*e*_ is then inferred from sibship frequencies in a sibship assignment analysis. A 95% confidence interval (C.I.) was calculated by assuming a t-student distribution. The second evaluation is the bias-corrected method based on LD [36–38] and implemented in NEESTIMATOR 2.1 [39]. It presumes that *N*_*e*_ is correlated to the degree of LD between two neutral markers in isolated populations of constant size and stable structure. To exclude low frequency and singletons alleles bias in the estimation, we did not use alleles that occur at a frequency less than 0.02 as recommended by Waples and Do [38]. We implemented the Jackknife-across-samples method for the 95% C.I. estimation. Random mating was assumed for both methods.

#### Bottlenecks

We used four methods to detect sharp population decline signatures. The first one, implemented in BOTTLENECK 1.2.02 [40], identifies a significant *H*_*O*_ excess compared to *H*_*E*_ for the number of observed loci [41]. We chose the two-phase mutation model (TPM) since it fits most microsatellite data sets compared to infinite allele or stepwise mutation models [42]. We set a 95% for single-step mutation, 5% for multiple step mutation, and the variance among multiple steps to 12. For statistical significance determination, a Wilcoxon sign-rank test was used [43]. The second method, which is also implemented in BOTTLENECK, is qualitative and indicates if the allele frequency distribution is approximately L-shaped, as a mutation-drift equilibrium expectation, or is shifted, as a recent bottleneck may cause [40]. The third method is the *M*-ratio test [44]. It assesses the relation between the number of alleles (k) and the overall range in fragment sizes (*r*), with the statistic *M =* k*/r+*1, as corrected by Excoffier and Lischer [31]. Since k is expected to decrease faster than *r* because of the loss of rare alleles by genetic drift, declining populations may have a smaller *M*-ratio than non-declining ones. This statistic was calculated in ARLEQUIN and established as significant if it was lower than a critical value (*M*_*c*_) obtained in simulations performed in CRITICAL_M [44]. We implemented the TPM model with an average repetition frequency of multi-step mutations *Δ*_*g*_ = 3.1, a proportion of multi-step mutations *p*_*g*_ = 0.22, as recommended by Peery and colleagues [45] to reduce I type error rates, and a *Ɵ* value defined as 4 *N*_*e*_*μ* (where *μ* = mutation rate) ranging from 0.1 to 10. Since the *M*-ratio test recovers from its signal much more slowly than the heterozygosity excess and the allele frequency distribution methods [44], a historical process might be detected if only the *M*-ratio is conclusive in favor of a population decline.

**Fig 2:**
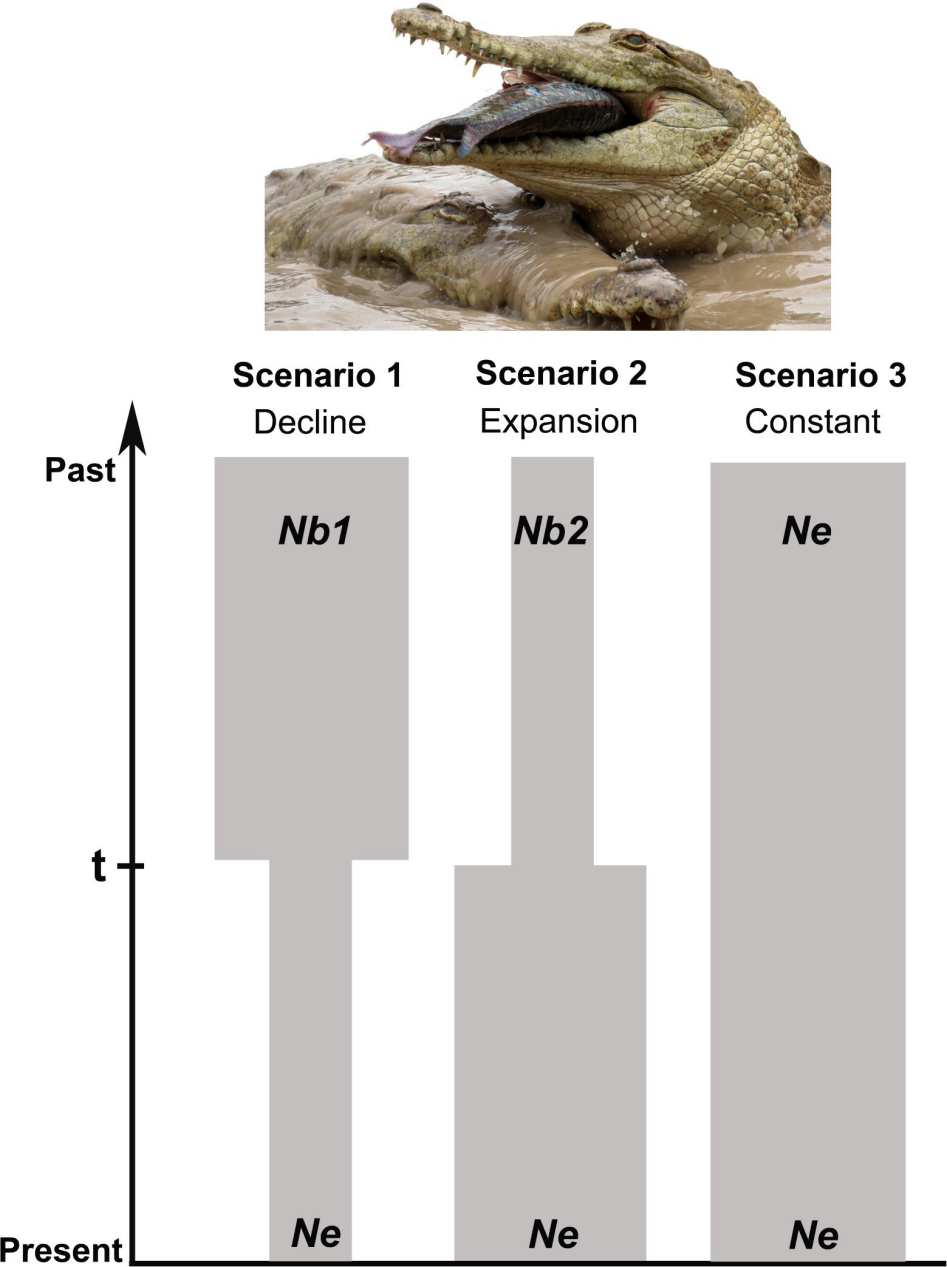
Demographic scenarios of decline, expansion, and constant effective population size assessed using Approximate Bayesian Computation (ABC) and coalescence simulations with DIYABC. *Ne* represents the contemporary effective population size; t is the time in generations when the population size changed; *Nb1* and *Nb2* represent the population size before the change. For simulating the models: *Nb1* > *Ne* for population decline, *Nb2* < *Ne* for population expansion, and *Ne* = *Ne* for a constant population size. In set photo: *Crocodylus intermedius* females at the EBTRF (Credit MVR).

However, the heterozygosity-excess tests, allele frequency distribution and *M*-ratio tests have limited power in detecting significant population bottlenecks, particularly when sampling occurs shortly after the bottleneck event [45]. Since this is the case for the sampling of the *C*. *intermedius* population, we also employed a fourth method: a coalescent-based Approximate Bayesian Computation (ABC) analyses using the software DIYABC v2.1.0 [46] to assess a very recent sharp population decline. DIYABC further allows for the evaluation of population size changes and the inference of demographic and historical parameters under the best-supported scenario. We compared three scenarios of population size changes: Scenario 1, a population decline; Scenario 2, a population expansion over time; and Scenario 3, a constant population size. In these scenarios, *Ne* represents the current population size, while *t* corresponds to the time in generations since the population size changed. *Nb1* and *Nb2* refer to the population sizes before the changes in Scenarios 1 and 2, respectively (Fig 2). We used the population dataset and simulated 9×10^6^ genetic datasets for all scenarios, assuming a generation time of 20 years [47]. To select the most likely scenario and infer parameter values under the best-supported scenario, we compared the real dataset with the simulated datasets using the following summary statistics: Mean number of alleles, mean genetic diversity, mean size variance, and mean Garza-Williamson’s *M* from One-sample summary statistics and mean number of alleles and mean genetic diversity from Two-sample summary statistics. Additionally, a uniform distribution was applied to all parameters. We conducted preliminary runs and adjusted the prior distributions for all parameters using the “prior checking” option. The resulting parameters were: *Ne* (10–10,000), *Nb1* (100–100,000), *Nb2* (10–10,000) and *t* (4–10,000). The best-supported scenario was identified by evaluating the relative posterior probability of each scenario using two methods: (1) a direct estimate and (2) a logistic regression approach. For the direct estimate, the 500 datasets with summary statistics closest to the observed data were selected from the nine million simulations. For the logistic regression approach, the top 1% (90,000) of the simulated datasets was used. The scenario with the highest significant posterior probability (P.P.) value and non-overlapping 95% C.I. was deemed the most likely. Additionally, the posterior distributions for the demographic parameters were estimated under the best-supported scenario using the top 1% of the best-fitting simulated datasets. By the inclusive application of these assessments, we may discern either a recent or a historical population decline.

#### Genetic diversity recovered by the hatchling ranching initiative

We inferred the same diversity indexes for the hatchling ranching initiative samples as the *in-situ* population and then compared their values. Observed allelic diversity (*A*_*Ob*_) and allelic richness (*A*_*R*_), were calculated using HP-RARE 1.0 [29]. Observed (*H*_*O*_) and expected heterozygosities (*H*_*E*_) were calculated using ARLEQUIN 3.5.1.2 [31].

## Results

### Population genetic variation

The loci Cj127 and CpP1610 were excluded from our analyses as they were found to be monomorphic. No significant linkage disequilibrium (LD) was detected between any pairs of loci, while locus CpP305 deviated significantly from HW equilibrium. Consequently, the subsequent analyses were conducted with the remaining 14 markers and 38 individuals (Table 1). Observed allelic diversity (*A*_*Ob*_) ranged from two (CpP3216 and CpP1409) to eight (C391) alleles, with a mean of 3.857 alleles per locus, while allelic richness (*A*_*R*_) ranged from 1.981 (CpP1409) to 5.575 (C391), with a mean of 3.109. The observed heterozygosity (*H*_*O*_) and expected heterozygosity (*H*_*E*_) values were 0.592 and 0.573, respectively. No null alleles were detected for any of the analyzed loci. Genotypes of the sampled individuals in the population are detailed in the S4 Table.

**Table 1:** Genetic diversity indexes and inbreeding coefficient (*F*_*IS*_) of microsatellite data for the *C*. *intermedius* population in the Cravo Norte-Ele-Lipa River System.

Locus	*A*_*Ob*_	*A*_*R*_	*H*_*O*_	*H*_*E*_	*F*_*IS*_
CpP3216	2.000	2.000	0.421	0.501	0.161
CpP1409	2.000	1.981	0.368	0.337	-0.095
CpP302	4.000	3.429	0.763	0.635	-0.206
CpP314	3.000	2.800	0.553	0.586	0.058
Cj16	4.000	3.422	0.526	0.501	-0.052
CU5123	4.000	3.201	0.632	0.602	-0.050
Cj122	5.000	4.688	0.684	0.775	0.118
Cj18	4.000	3.932	0.842	0.739	-0.142
CUJ131	3.000	2.104	0.211	0.195	-0.080
Cj109	3.000	2.980	0.684	0.640	-0.070
C391	8.000	5.575	0.816	0.804	-0.015
Cj101	4.000	2.555	0.658	0.543	-0.216
CpDi13	3.000	2.197	0.526	0.488	-0.080
CpP801	5.000	3.802	0.605	0.681	0.113
**Mean**	**3.857**	**3.190**	**0.592**	**0.573**	**-0.040**
s.d.	1.512	1.060	0.175	0.166	0.116

*A*_*Ob*_ observed allelic diversity; *A*_*R*_ allelic richness; *H*_*O*_ observed heterozygosity; *H*_*E*_ expected heterozygosity.

### Inbreeding and effective population size

The estimated mean F_IS_ value was -0.040 (Table 1), and it did not show any statistically significant deviation for either excess or deficiency of heterozygotes evaluated per loci and population. Consequently, there is no evidence of current inbreeding. However, both estimations of effective population size (*Ne*) indicated a concerning value for this parameter: 17 (95% C.I. = 10.0–34.0) by sibship assignment and 11.5 (95% C.I. = 5.7–21.7) by linkage disequilibrium.

### Bottlenecks

A recent bottleneck was not detected using either the heterozygosity excess method or the allele frequency distribution approach. However, the Wilcoxon signed-rank test applied in the heterozygosity excess method yielded a *p* value of 0.059, which is close to the statistical significance threshold (Table 2). In contrast, the analysis using DIYABC supported the presence of a decline in the population. Scenario 1, which proposed that the population had undergone a population decline, was strongly supported by both the direct-estimate approach (P.P. = 0.95, C.I. = 0.74–1.00) and the logistic regression approach (P.P. = 0.91, C.I. = 0.90–0.92). In comparison, Scenario 2 (population expansion) and Scenario 3 (constant population size) received little to no support. For the direct-estimate approach, P.P. and C.I. were 0.01 (0–0.1) and 0.05 (0–0.3), respectively. For the logistic regression approach, P.P. and C.I. were 0.02 (0.02–0.03) and 0.06 (0.05–0.06), respectively (S1 Fig). Principal component analysis (PCA) plots of the summary statistics confirmed that the observed data fell within the range of the simulated data (S2 Fig). Additionally, the model-checking plot demonstrated that the model/posterior accurately explained the observed dataset (S3 Fig).

**Table 2:** Assessment of bottlenecks in the *in-situ* population of *C*. *intermedius*.

Wilcoxon sign-rank test (one-tailed *p* value for heterozygosity excess)	Allele frequency distribution mode shift test	*M*-ratio test		DIYABC analysis
0.059	Normal L-shaped	*M*-ratio	*M*_*c*_	Historical population decline
		0.365*	0.679–0.802	

* statistically significant.

However, the inferred parameter for the posterior distribution of *t* indicated that the population size declined approximately 25,400 years ago (1,270 generations; C.I. = 196–7,410). This analysis identified a historical population declined rather than the more recent one known to have occurred in the last century. The estimated strength of this historical decline was an approximately 4-fold reduction in population size, with *Nb1* = 8,490 (C.I. = 3,580–9,890) and *Ne* = 1,750 (C.I. = 707–4,270) (S4 Fig). Evidence of population decline was also detected using the *M*-ratio test, with consistent results across different θ values for the *Mc* threshold (Table 2). These findings are consistent with the DIYABC analyses, further supporting the occurrence of a historical decline in the population.

### Genetic diversity recovered by the hatchling ranching initiative

Within the conservation dataset, loci Cj127 and CpP1610 were identified as monomorphic and, consequently, excluded from the analyses. Locus CpP305 was also excluded as it deviated significantly from HW equilibrium. Table 3 displays the diversity indexes for the 81 samples derived from the hatchling ranching initiative, while the S5 Table provides their corresponding genotypes. A marginal reduction in the mean value for each index was observed compared to those identified for the *in-situ* population in Table 1. Allelic diversity (*A*_*Ob*_) ranged from two (CpP3216, CpP1409, and CpDi13) to eight (C391) alleles, with a mean of 3.714 alleles per locus, signifying a 3.7% reduction from the population value. Allelic richness (*A*_*R*_) ranged from 1.860 (CpP1409) to 5.602 (C391), with a mean of 3.165, indicating a reduction of 0.8% from the population value. Notably, for locus Cj109, one additional allele (allele 382) was identified compared to the population dataset. Concerning heterozygosity measures, observed heterozygosity (*H*_*O*_) demonstrated a value of 0.574, denoting a 3% reduction from the population value. Expected heterozygosity (*H*_*E*_) was 0.569, representing a 0.7% reduction from the population value. These findings highlight a subtle decrease in genetic diversity in the samples from the hatchling ranching initiative compared to the *in-situ* population.

**Table 3:** Genetic diversity indexes for the conservation dataset.

Locus	*A*_*Ob*_	*A*_*R*_	*H*_*O*_	*H*_*E*_
CpP3216	2	1.989	0.469	0.374
CpP1409	2	1.860	0.235	0.208
CpP302	4	3.483	0.654	0.590
CpP314	3	2.920	0.704	0.619
Cj16	4	3.191	0.519	0.489
CU5123	3	2.858	0.556	0.574
Cj122	5	4.347	0.642	0.735
Cj18	4	3.856	0.815	0.707
CUJ131	3	2.656	0.370	0.462
Cj109	4	3.064	0.556	0.647
C391	8	5.602	0.877	0.808
Cj101	4	2.566	0.482	0.544
CpDi13	2	2.000	0.407	0.478
CpP801	4	3.914	0.753	0.737
**Mean**	**3.714**	**3.165**	**0.574**	**0.569**
s.d.	1.541	1.027	0.179	0.161
**Reduction**	**3.7%**	**0.8%**	**3%**	**0.7%**

Reduction indicates the percentage by which each index has decreased compared to the values identified for the *in-situ* population.

## Discussion

### Population genetic status

The population dataset used to infer the genetic diversity parameters in this research spans eight years (2009–2017). Given that crocodilians are known for they long generation times–estimated at around 20 years for *Crocodylus* species [47]–we contend that this sampling period provides a valid representation of the population’s genetic status. The inferred parameters indicate that the Orinoco Crocodile population residing in the Cravo Norte-Ele-Lipa River System exhibits moderated levels of genetic diversity. In the S6 Table, we present previous estimations performed with microsatellites in *Crocodylus*. These estimates provide a basis for comparing the results obtained in this study. Nonetheless, because comparisons between different genomic regions can lead to imprecise conclusions, the contrast made here should be considered as approximate. The reported heterozygosity indexes (*H*_*O*_ = 0.592 and *H*_*E*_ = 0.573) are slightly higher than those observed in other Critically Endangered *Crocodylus* species, such as *C*. *mindorensis* (*H*_*O*_ = 0.408 to 0.457, *H*_*E*_ = 0.423 to 0.446; [48]; S6 Table) and *C*. *rhombifer* (*H*_*O*_ = 0.490, *H*_*E*_ = 0.540; [49]; S6 Table). Additionally, the revealed genetic diversity values are within the range and, in some cases, slightly higher than those of other crocodile species categorized as Vulnerable or even of Least Concern (see S6 Table). On the contrary, the mean number of alleles per locus (*A*_*Ob*_ = 3.857) is notably lower than the reported median (S6 Table).

The genetic diversity indexes observed in the evaluated *in-situ* population are lower than those found in *ex-situ* populations of the species in terms of *A*_*Ob*_ and *A*_*R*_ (S6 Table). This result is expected, as *ex-situ* populations consist of individuals from various locations within the species’ distribution range [24, 25]. In comparison to the described genetic clusters of the Orinoco Crocodile in the Colombian Orinoquía [26], our evaluation reveals higher estimations of *H*_*O*_ in the Cravo Norte-Ele-Lipa population (S6 Table). Consequently, we assert that the population assessed in this study represents a genetically valuable resource for the conservation of the species. However, the low estimates of *A*_*Ob*_ and *A*_*R*_ may suggest a potential process of genetic diversity loss. In population declines, such as those experienced by the species in the last century due to anthropogenic actions [3, 36], alleles are lost more rapidly than heterozygosity [43].

The results obtained for contemporary *Ne* estimations reveal an exceedingly low value for this parameter in the evaluated Orinoco Crocodile population. When *Ne* was first recognized as a valuable tool in conservation management, the 50–500 rule was proposed: a population should ideally maintain a *Ne* of at least 50 to mitigate the risk of short-term extinction caused by inbreeding depression [50]. Moreover, a minimum *Ne* of 500 is deemed necessary to strike a balance between genetic drift and mutation, ensuring the retention of sufficient genetic variation for long-term adaptation [50]. Recent perspectives advocate for revised minimum values of *Ne*, suggesting *Ne* ≥ 100 and *Ne* ≥ 1000 for short-term and long-term conservation, respectively [51]. However, ongoing discussions persist regarding whether *Ne* should be set at 50 or 100 for short-term conservation [52]. Regardless of this debate, the upper bound of the *Ne* estimated for the *C*. *intermedius* population in the Cravo Norte-Ele-Lipa River System, with a value of 34.0 identified by the sibship assignment method, falls below any proposed value for short-term subsistence. This alarming scenario likely mirrors the population’s recent demographic history of intense hunting, underscoring the profound impact on even Colombia’s presumably largest remaining Orinoco Crocodile wild population.

The current high genetic diversity and the absence of clear evidence for inbreeding may suggest that the population is not currently in the extinction vortex [53]. However, the inferred *Ne* values underscore the potential risk of initiating an inbreeding depression process, which could lead to reduced genetic diversity and fitness [54]. Indeed, the sharp recent population decline due to extreme hunting in this population has likely resulted in a very recent bottleneck (not identified by our analyses but see below). Recent bottlenecks might affect F_IS_ values, underestimating the proper level of inbreeding. Such demographic processes temporarily elevate *H*_*O*_ relative to *H*_*E*_, resulting from losing low-frequency alleles [55, 56]. Consequently, we cannot rule out the possibility that the evaluated population has already initiated an inbreeding process. Hinlo and colleagues [48] reported a similar case for the Critically Endangered *C*. *mindorensis* wild populations, with even lower *Ne* estimates and no evidence of inbreeding. However, unlike *C*. *intermedius*, this species presented low genetic diversity measured in terms of *H*_*E*_ (0.423–0.446; [48]; S6 Table).

### Demographic history

*Crocodylus intermedius* is a long-lived species, and historical records indicate that its populations were once abundant. For instance, Alexander von Humboldt documented numerous encounters with this crocodile during his exploration of the Orinoco River and its tributaries in the early 19th century and how the species was an integral part of the daily life of human communities inhabiting the region [57].

Despite limited information, Medem [3] estimated an average export of 90,000 skins per year between 1930 and 1970, while other sources propose a minimum of two to three million exports during that period [4, 58]. This extensive exploitation led to a significant population decline of the species [3, 4, 58, 59]. However, our analyses did not distinctly reveal a recent anthropogenically induced sharp population decline; instead, they point towards a historical decline. Regarding the heterozygosity excess method, theoretical models indicate that this process is detectable for a window of time after the population reduction occurred; for instance, the signal of a bottleneck of *Ne* = 50 may remain for a period of 0.2–2.5 *Ne* after a population reduction [41]. Likewise, a mode-shifted distribution may take five to ten generations to manifest with 20 breeders, persisting for a few dozen generations [43].

Although coalescent-based ABC analyses have been used to detect very recent population declines in endangered species using microsatellite data [60, 61], in this case, they identified a signal of a historical decline but failed to detect the recent decline known to have occurred between the 1920s and 1960s. The *M*-ratio test maintains a signal for more than 100 generations [44]. Considering the species’ longevity, the recent sharp population decline experienced by the Orinoco Crocodile might be too recent to be detected using heterozygosity excess, mode-shift distribution, or ABC approximations. A similar scenario was observed in the Critically Endangered gharial (*Gavialis gangeticus*) in India, where the heterozygosity excess method did not identify the dramatic population decline in the last century [62].

The results of the coalescent-based ABC analysis indicated that the population experienced a sharp decline approximately 25,400 years ago (C.I. = 3,900–148,000 years ago), reducing its size from an ancestral effective population size (*Nb1*) of 8,490 (C.I. = 3,580–9,890) to a post-decline effective population size (*Ne*) of 1,750 (CI: 707–4,270). A biogeographically significant process that may explain the detected population decline is the historical changes affecting hydrological and habitat conditions in the Orinoquía. The pollen record indicates that this region experienced an arid period with lower precipitation and extended dry seasons between the Last Glacial Maximum (20,000 to 18,000 years ago) and the late Pleistocene (13,000 to 10,000 years ago) [63, 64]. Additionally, it has been proposed that the aeolian savannas characterizing the region where the Cravo Norte-Ele-Lipa River System is situated, originated from a desertic climate in the Late Pleistocene [65, 66]. As a result, during that period, the region’s hydrology experienced a diminished discharge of the Orinoco River [66]. Given that *C*. *intermedius* demonstrates a high degree of dependence on aquatic environments–being a riverine species confined to the water systems in the Orinoco [67] that prefer distant areas from the shore [68]–it is highly plausible that this process influenced the population decline we are observing by diminishing the habitat quality essential for the species. Reductions in effective population sizes due to glacial cycles of the Pleistocene have already been documented in other crocodilian species [47]. Additionally, it may have been possible that arid periods during the early and middle Holocene may have contributed to similar scenarios [63, 64, 66].

### Conservation implications

This research marks a significant advancement in the conservation efforts for *C*. *intermedius* and addresses a crucial aspect in the ongoing initiatives to prevent the extinction of the species in Colombia, such as PROCAIMAN [18] and the Interinstitutional Action Plan for the Orinoco Crocodile Conservation [8]. It represents a significant progress in the genetic evaluation of wild populations. The crocodile population in the Cravo Norte-Ele-Lipa River System stands out as one of Colombia’s most studied wild populations of the species. Apart from the genetic evaluation presented in this research, there exists data regarding its abundance and ecology [9–11, 13, 69]. Additionally, there are promising conservation initiatives involving the local human community. Despite adult crocodile killings and nest harvesting [8,12], the community has expressed a keen interest in contributing to conserving this emblematic species [8, 70]. These factors, the acceptable evolutionary potential identified, coupled with indications of a population recovery process [13, 69], present a unique and promising opportunity for the long-term survival of *C*. *intermedius*.

Despite the considerable human pressures endured by the species over the last century, a discernible genetic signature persists in this Orinoco Crocodile population. This is evident in detecting a reduced *Ne* as a likely consequence of its recent population decline. Nevertheless, the population still retains a significant genetic diversity reservoir, similar to that observed in other wild populations of non-threatened crocodiles. Additionally, our analysis indicates that the hatchling ranching initiative implemented in 2016 successfully contributed to the recovery of much of the population’s diversity (Table 3). Although it focused on clutches from a single sand beach in the Cravo Norte River, expanding the initiative to include clutches from other areas within the evaluated River System is recommended. The recent increase in reported nest locations [13] supports the feasibility of this approach, promising further enhancement of the diversity recovered by the conservation program. Therefore, given the revealed evolutionary potential of this population and the positive observations concerning other aspects of the Orinoco Crocodiles in the area, we propose that conservation efforts for this population should prioritize demographic increase through the involvement of local individuals, employing ranching strategies.

On the other hand, it is crucial to assess the usefulness of population reinforcement to increase the number of adult parental pairs based on the management units proposed for the species by Castillo-Rodríguez and colleagues [26]. However, potential risks, such as disease transmission and the impact on learned behaviors, should be carefully considered. If population reinforcement is pursued, it is advisable to prioritize specimens from the *ex-situ* population of the EBTRF whose genetic identity aligns primarily with the Northern Management Unit located in the Eastern Meta River basin, as proposed by Castillo-Rodríguez and colleagues [26], given that the evaluated population is part of this unit. Moreover, considering that population fragmentation poses a significant threat to the preservation of genetic diversity and that gene flow between isolated populations can substantially increase *Ne* [51, 71], evaluating the possibility of a genetic rescue introducing individuals with identity from the Central Management Unit is essential. There is evidence of limited gene flow between populations within the Eastern Meta basin (Northern Management Unit) and the Western Meta Basin and Vichada Basin (Central Management Unit) (*m* = 0.052–0.089; [26]). This can simulate a process of migration that likely occurred more extensively in the past, aiming to preserve the identified genetic diversity and increase the currently depleted *Ne*. The *ex-situ* population of the EBTRF possesses adult individuals that can be used for this purpose [25]. This initiative would only accelerate a process that could occur naturally since both units are effectively connected, albeit distant, through the intricate hydrological network of the region.

On the other hand, even if the risk of outbreeding depression is lower than was previously thought [72, 73], it might be elevated if populations had been isolated for more than 500 years or inhabit different environments [72]. As a consequence, reinforcements with individuals of the Guaviare Basin population (Southern Management Unit of Castillo-Rodríguez and colleagues [26]) must be avoided since it inhabits an area characterized by riparian and floodable forests, while the Cravo Norte-Ele-Lipa River System is characterized by floodplains ecosystems [74], and presents a distinctive genetic identity [26] and most probably an adaptive separated group.

Finally, it is necessary to survey the genetic status of the population continuously to i) maintain its genetic diversity, ii) revise for changes in its *Ne*, and iii) opportunely identify inbreeding. As Jamieson and Allendorf [75] discussed, conservation programs’ goals should emphasize maintaining genetic diversity during the recovery stage, and not solely reaching a minimum recovery size and implying that an *Ne* that might guarantee the population’ viability must be a long-term aspirational purpose.

## Conclusions

We have identified crucial aspects of the remnant population of *C*. *intermedius* inhabiting the Cravo Norte-Ele-Lipa River System. The population exhibits moderate levels of genetic diversity. However, estimates of allele richness are relatively low, suggesting a potential process of genetic diversity loss. Critical demographic factors have been determined for consideration in management actions, including an extremely low effective population size and the apparent absence of inbreeding. This indicates that, while the risk is high, the population is not currently in the extinction vortex. Nevertheless, it’s possible that inbreeding is not detectable due to the proximity of the sharp population decline.

Furthermore, we emphasize the significance of historical ecological and hydrogeographic processes in the species’ population history. Reductions in the Orinoquía Rivers level during the Late Pleistocene might have induced a population decline, as detected in the Cravo Norte-Ele-Lipa River System population. The results presented in this research support demographic increase with local individuals as the primary conservation action for the evaluated Orinoco Crocodile population. Therefore, we advocate for the egg and hatchling ranching initiatives in this and other populations, which have successfully recovered genetic diversity. Lastly, we emphasize the importance of continuing and strengthening Orinoco Crocodile management by integrating genetic, ecological, and anthropological perspectives. This is fundamental in aiming not just for a scientific point of view to improve our knowledge of the species and its habitat but also involving the co-existing human community, whose role is directly responsible for the real success of any conservation program.

## Supporting information

S1 Table Sampled individuals from the Cravo Norte-Ele-Lipa River System in Arauca, Colombia. (DOCX)

S2 Table Sampled individuals from the hatchling ranching program in the Cravo Norte municipality, Arauca, Colombia. (DOCX)

S3 Table Set of primers used in this study. (DOCX)

S4 Table Genotypes of *C*. *intermedius* individuals analyzed in the *in-situ* population genetics assessment. (DOCX)

S5 Table Genotypes of the *C*. *intermedius* individuals analyzed in the Cravo Norte ranching program genetics assessment. (DOCX)

S6 Table Genetic diversity parameters of populations of *C*. *intermedius* and other wild crocodile populations. Previous *C*. *intermedius* evaluations for *ex-situ* populations are indicated with an asterisk. *N* sample size; *H*_*O*_ observed heterozygosity; *H*_*E*_ expected heterozygosity; *A*_*Ob*_ observed allelic diversity; *A*_*R*_ allelic richness. IUCN categories are NE Not Evaluated; LC Least Concerned; VU Vulnerable; CR Critically Endangered.
(DOCX)

S1 Fig Plots showing the fit of three tested demographic scenarios, based on direct estimates and logistic regression, simulated in DIYABC. Note the strong support for Scenario 1; population decline. For the parameter settings in each scenario, refer to Materials and Methods and Fig 2.
(TIF)

S2 Fig Distribution of simulated plots for three alternative scenarios alongside the observed data. It confirms that the model fits well, as the genetic data fall within the range of the simulated results.
(TIF)

S3 Fig Model checking plot. It shows the observed data set summary statistics value and proportion of datasets (simulated from the posterior) that have a value lower than the observed dataset.
(TIF)

S4 Fig Prior and posterior distributions density curves calculated under Scenario 1 for *C*. *intermedius* in DIYABC. Times are not scaled.
(TIF)

## References

[1] Seijas AE, Antelo R, Thorbjarnarson JB, Robayo MCA. Orinoco Crocodile Crocodylus intermedius. Third Edit. In: Manolis SC, Stevenson C, editors. Crocodiles Status Survey and Conservation Action Plan. Third Edit. Darwin: Crocodile Specialist Group; 2010. pp. 59–65.

[2] Balaguera-Reina SA, Espinosa-Blanco AS, Morales-Betancourt MA, Seijas AE, Lasso CA, Antelo R, et al. Conservation status and regional habitat priorities for the Orinoco crocodile: Past, present, and future. PLoS One. 2017;12: e0172439. doi: 10.1371/journal.pone.0172439 28234956 PMC5325271

[3] Medem F. Los Crocodylia de Sur America: Los Crocodylia de Colombia. Vol. 1. Bogotá: Ministerio de Educacion Nacional, Fondo Colombiano de Investigaciones Científicas y Proyectos Especiales “Francisco José de Caldas”; 1981.

[4] Antelo R. Biología del cocodrilo o caimán del Orinoco (Crocodylus intermedius) en la Estación Biológica El Frío, Estado Apure. D. Phil. Thesis, Universidad Autónoma de Madrid. Universidad Autónoma de Madrid. 2008.

[5] Balaguera-Reina SA, Espinosa-Blanco A, Antelo R, Morales-Betancourt M, Seijas A. Crocodylus intermedius (errata version published in 2020). In: The IUCN Red List of Threatened Species 2018: e.T5661A181089024. 2018. doi: 10.2305/IUCN.UK.2018-1.RLTS.T5661A181089024.en

[6] CITES. Appendices I, II and III valid from 25 November 2023. CITES-UNEP. 2023. Available: https://cites.org/sites/default/files/eng/app/2023/E-Appendices-2023-11-25.pdf

[7] Castro A, Merchán M, Gómez F, Garcés MF, Cárdenas MA. Nuevos datos sobre la presencia de caimán llanero (Crocodylus intermedius) y notas sobre su comportamiento en el río Vichada, Orinoquia (Colombia). Biota Colomb. 2011;12: 137–144. doi: 10.21068/bc.v12i1.244

[8] Antelo R, Vargas-Ramírez M, Preciado G, Saavedra-Rodríguez CA, Forero-Medina G. Plan de acción interinstitucional para la conservación del caimán llanero (Crocodylus intermedius) en Colombia. Cali: Wildlife Conservation Society, Estación de Biología Tropical Roberto Franco, Gobernación de Casanare, Universidad Nacional; 2022.

[9] Lugo-Rugeles LM, Ardila-Robayo MC. Programa para la conservación del caiman del Orinoco (Crocodylus intermedius) en Colombia. Proyecto 290. Programa Research Fellowship NYZS. Wildlife Conservation Society. Proyecto 1101-13- 205–92 Colciencias. Villavicencio; 1998.

[10] Ardila-Robayo MC, Barahona-Buitrago SL, Bonilla-Centeno OP, Clavijo BJ. Actualización del status poblaciones de Caimán del Orinoco (Crocodylus intermedius) en el Departamento de Arauca (Colombia). In: Velasco A, Colomine G, Villarroel G, Quero M, editors. Memorias del Taller para la Conservación del Caimán del Orinoco (Crocodylus intermedius) en el Colombia y Venezuela. 2002. pp. 57–67.

[11] Barahona-Buitrago SL, Bonilla-Centeno OP. Evaluación poblacional del Caimán Llanero (Crocodylus intermedius) en un subareal de distribución en el departamento de Arauca (Colombia). Rev la Acad Colomb Ciencias. 1999;23: 445–451.

[12] Castro A, Merchán M, Garcés M, Cárdenas M, Gómez F. New data on the Conservation Status of the Orinoco crocodile (Crocodylus intermedius) in Colombia. Crocodiles Proceedings of the 21st WorkingMeeting of the IUCN-SSC Crocodile Specialist Group. Gland: IUCN; 2012. pp. 65–73.

[13] Anzola LF, Antelo R. First data of natural recovery of any Orinoco crocodile Crocodylus intermedius population: Evidence from nesting. Herpetol Bull. 2015; 10–14.

[14] Willi Y, Kristensen TN, Sgro CM, Weeks AR, Ørsted M, Hoffmann AA. Conservation genetics as a management tool: The five best-supported paradigms to assist the management of threatened species. Proc Natl Acad Sci U S A. 2022;119: 1–10. doi: 10.1073/pnas.2105076119 34930821 PMC8740573

[15] Jamieson IG, Grueber CE, Waters JM, Gleeson DM. Managing genetic diversity in threatened populations: a New Zealand perspective. N Z J Ecol. 2008;32: 130–137.

[16] Reed DH, Frankham R. Correlation between fitness and genetic diversity. Conserv Biol. 2003;17: 230–237. doi: 10.1046/j.1523-1739.2003.01236.x

[17] Vandewoestijne S, Schtickzelle N, Baguette M. Positive correlation between genetic diversity and fitness in a large, well-connected metapopulation. BMC Biol. 2008;6: 1–11. doi: 10.1186/1741-7007-6-46 18986515 PMC2587462

[18] MMA. Programa Nacional para la Conservación del Caimán Llanero (PROCAIMÁN). Bogotá: Ministerio del Medio Ambiente Dirección General de Ecosistemas Subdirección de Fauna; 2002. p. 31.

[19] QGIS.org. QGIS Geographic Information System. Open Source Geospatial Foundation Project; 2024. Available: http://qgis.org

[20] Lehner B, Grill G. Global river hydrography and network routing: baseline data and new approaches to study the world’s large river systems. Hydrol Process. 2013;27: 2171–2186. doi: 10.1002/hyp.9740

[21] Fitzsimmons NN, Tanksley S, Forstner MRJ, Louis EE, Daglish R, Gratten J, et al. Microsatellite markers for Crocodylus: new genetic tools for population genetics, mating system studies and forensics. In: Grigg G, Seebacher F, Franklin CE, editors. Conference on Crocodilian Biology and Evolution. St Lucia Australia: Surrey Beatty & Sons; 2001. pp. 51–57.

[22] Dever JA, Densmore LD. Microsatellites in Morelet’s Crocodile (Crocodylus moreletii) and Their Utility in Addressing Crocodilian Population Genetics Questions. J Herpetol. 2001;35: 541–544.

[23] Miles LG, Isberg SR, Moran C, Hagen C, Glenn TC. 253 Novel polymorphic microsatellites for the saltwater crocodile (Crocodylus porosus). Conserv Genet. 2009;10: 963–980. doi: 10.1007/s10592-008-9600-7

[24] Rossi Lafferriere NA, Antelo R, Alda F, Martensson D, Hailer F, Castroviejo-Fisher S, et al. Multiple paternity in a reintroduced population of the orinoco crocodile (crocodylus intermedius) at the El frío biological station, Venezuela. PLoS One. 2016;11: e0235288. doi: 10.1371/journal.pone.0150245 26982578 PMC4794145

[25] Saldarriaga-Gómez AM, Ardila-Robayo MC, Medem F, Vargas-Ramírez M. Hope is the last thing lost: Colombian captive-bred population of the critically endangered Orinoco crocodile (Crocodylus intermedius) is a genetic reservoir that could help to save the species from extinction. Nat Conserv. 2023;103: 85–103. doi: 10.3897/natureconservation.53.104000

[26] Castillo-Rodríguez N, Saldarriaga-Gómez AM, Antelo R, Vargas-Ramírez M. Population genetic structure in the critically endangered Crocodylus intermedius (Crocodilia: Crocodylidae): a shift in perspective for conservation actions in Colombia. Biol J Linn Soc. 2024; 1–15. doi: 10.1093/biolinnean/blad174

[27] van Oosterhout C, Hutchinson WF, Wills DPM, Shipley P. Micro-Checker: software for identifying and correcting genotyping errors in microsatellite data. Mol Ecol Notes. 2004;4: 535–538. doi: 10.1111/j.1471-8286.2004.00684.x

[28] Rousset F. GENEPOP ‘ 007: a complete re-implementation of the GENEPOP software for Windows and Linux. Mol Ecol Resour. 2008;8: 103–106. doi: 10.1111/j.1471-8286.2007.01931.x 21585727

[29] Kalinowski ST. HP-RARE 1.0: A computer program for performing rarefaction on measures of allelic richness. Mol Ecol Notes. 2005;5: 187–189. doi: 10.1111/j.1471-8286.2004.00845.x

[30] Goudet J. FSTAT (Version 1.2): A Computer Program to Calculate F-Statistics. J Hered. 1995;86: 485–486. doi: 10.1093/oxfordjournals.jhered.a111627

[31] Excoffier L, Lischer HEL. Arlequin suite ver 3.5: a new series of programs to perform population genetics analyses under Linux and Windows. Mol Ecol Resour. 2010;10: 564–567. doi: 10.1111/j.1755-0998.2010.02847.x 21565059

[32] Weir BS, Cockerham CC. Estimating F-Statistics for the Analysis of Population Structure. Evolution (N Y). 1984;38: 1358–1370. doi: 10.1111/j.1558-5646.1984.tb05657.x 28563791

[33] Rousset F, Raymond M. Testing heterozygote excess and deficiency. Genetics. 1995;140: 1413–1419. doi: 10.1093/genetics/140.4.1413 7498780 PMC1206704

[34] Wang J. A new method for estimating effective population sizes from a single sample of multilocus genotypes. Mol Ecol. 2009;18: 2148–2164. doi: 10.1111/j.1365-294X.2009.04175.x 19389175

[35] Jones OR, Wang J. COLONY: A program for parentage and sibship inference from multilocus genotype data. Mol Ecol Resour. 2010;10: 551–555. doi: 10.1111/j.1755-0998.2009.02787.x 21565056

[36] Hill WG. Estimation of effective population size from data on linkage disequilibrium. Genet Res. 1981;38: 209–216. doi: 10.1017/S0016672300020553

[37] Waples RS. A bias correction for estimates of effective population size based on linkage disequilibrium at unlinked gene loci. Conserv Genet. 2006;7: 167–184. doi: 10.1007/s10592-005-9100-y

[38] Waples RS, Do C. Linkage disequilibrium estimates of contemporary Ne using highly variable genetic markers: A largely untapped resource for applied conservation and evolution. Evol Appl. 2010;3: 244–262. doi: 10.1111/j.1752-4571.2009.00104.x 25567922 PMC3352464

[39] Do C, Waples RS, Peel D, Macbeth GM, Tillett BJ, Ovenden JR. NeEstimator v2: re-implementation of software for the estimation of contemporary effective population size (Ne) from genetic data. Mol Ecol Resour. 2014;14: 209–214. doi: 10.1111/1755-0998.12157 23992227

[40] Piry S, Luikart G, Cornuet J-M. Computer note. BOTTLENECK: a computer program for detecting recent reductions in the effective size using allele frequency data. J Hered. 1999;90: 502–503. doi: 10.1093/jhered/90.4.502

[41] Cornuet JM, Luikart G. Description and power analysis of two tests for detecting recent population bottlenecks from allele frequency data. Genetics. 1996;144: 2001–2014. doi: 10.1093/genetics/144.4.2001 8978083 PMC1207747

[42] Di Rienzo A, Peterson AC, Garza JC, Valdes AM, Slatkin M, Freimer NB. Mutational processes of simple-sequence repeat loci in human populations. Proc Natl Acad Sci U S A. 1994;91: 3166–3170. doi: 10.1073/pnas.91.8.3166 8159720 PMC43536

[43] Luikart G, Cornuet J-M. Empirical evaluation of a test for identifying recently bottlenecked populations from allele frequency data. Conserv Biol. 1998;12: 228–237. doi: 10.1111/j.1523-1739.1998.96388.x

[44] Garza JC, Williamson EG. Detection of reduction in population size using data from microsatellite loci. Mol Ecol. 2001;10: 305–318. doi: 10.1046/j.1365-294x.2001.01190.x 11298947

[45] Peery MZ, Kirby R, Reid BN, Stoelting R, Doucet-Bëer E, Robinson S, et al. Reliability of genetic bottleneck tests for detecting recent population declines. Mol Ecol. 2012;21: 3403–3418. doi: 10.1111/j.1365-294X.2012.05635.x 22646281

[46] Cornuet J-M, Pudlo P, Veyssier J, Dehne-Garcia A, Gautier M, Leblois R, et al. DIYABC v2.0: a software to make approximate Bayesian computation inferences about population history using single nucleotide polymorphism, DNA sequence and microsatellite data. Bioinformatics. 2014;30: 1187–1189. doi: 10.1093/bioinformatics/btt763 24389659

[47] Green RE, Braun EL, Armstrong J, Earl D, Nguyen N, Hickey G, et al. Three crocodilian genomes reveal ancestral patterns of evolution among archosaurs. Science. 2014;346: 1254449. doi: 10.1126/science.1254449 25504731 PMC4386873

[48] Hinlo MRP, Tabora JAG, Bailey CA, Trewick S, Rebong G, van Weerd M, et al. Population genetics implications for the conservation of the Philippine Crocodile Crocodylus mindorensis Schmidt, 1935 (Crocodylia: Crocodylidae). J Threat Taxa. 2014;6: 5513–5533. doi: 10.11609/jott.o3384.5513–33

[49] Milián-García Y, Ramos-Targarona R, Pérez-Fleitas E, Sosa-Rodríguez G, Guerra-Manchena L, Alonso-Tabet M, et al. Genetic evidence of hybridization between the critically endangered Cuban crocodile and the American crocodile: implications for population history and in situ/ex situ conservation. Heredity. 2015;114: 272–280. doi: 10.1038/hdy.2014.96 25335559 PMC4815585

[50] Franklin IR. Evolutionary changes in small populations. In: Soulé ME, Wilcox BA, editors. Conservation biology: an evolutionary-ecological prospective. Sunderland: Sinauer Associates; 1980. pp. 135–150.

[51] Frankham R, Bradshaw CJA, Brook BW. Genetics in conservation management: Revised recommendations for the 50/500 rules, Red List criteria and population viability analyses. Biol Conserv. 2014;170: 56–63. doi: 10.1016/j.biocon.2013.12.036

[52] García-Dorado A. On the consequences of ignoring purging on genetic recommendations for minimum viable population rules. Heredity (Edinb). 2015;115: 185–187. doi: 10.1038/hdy.2015.28 25873145 PMC4814235

[53] Gilpin M, Soulé ME. Minimum viable populations: Processes of species extinction. In: Soulé ME, Wilcox BA, editors. Conservation biology: an evolutionary-ecological prospective. Sunderland: Sinauer Associates; 1986. pp. 19–34.

[54] Blomqvist D, Pauliny A, Larsson M, Flodin LÅ. Trapped in the extinction vortex? Strong genetic effects in a declining vertebrate population. BMC Evol Biol. 2010;10: 1–9. doi: 10.1186/1471-2148-10-33 20122269 PMC2824661

[55] van Asch B, Versfeld WF, Hull KL, Leslie AJ, Matheus TI, Beytell PC, et al. Phylogeography, genetic diversity, and population structure of Nile crocodile populations at the fringes of the southern African distribution. PLoS One. 2019;14: e0226505. doi: 10.1371/journal.pone.0226505 31869351 PMC6927622

[56] Rhode C, Maduna SN, Roodt-Wilding R, Bester-Van Der Merwe AE. Comparison of population genetic estimates amongst wild, F1 and F2 cultured abalone (Haliotis midae). Anim Genet. 2014;45: 456–459. doi: 10.1111/age.12142 24617992

[57] von Humboldt A. Vom Orinoko zum Amazonas: Reise in die Äquinoktial-Gegenden des neuen Kontinents. Plott A, editor. Frankfurt am Main: F. A. Brockhaus; 1958.

[58] Thorbjarnarson JB. Status, ecology and conservation of the Orinoco Crocodile. Preliminary Report. Caracas: FUDENA-WWF; 1987. p. 74.

[59] Casal AC, Fornelino MM, Restrepo MFG, Torres MAC, Velasco FG. Uso histórico y actual del caimán llanero (Crocodylus intermedius) en la Orinoquia (Colombia-Venezuela). Biota Colomb. 2013;14: 65–82.

[60] Tison J-L, Blennow V, Palkopoulou E, Gustafsson P, Roos A, Dalén L. Population structure and recent temporal changes in genetic variation in Eurasian otters from Sweden. Conserv Genet. 2015;16: 371–384. doi: 10.1007/s10592-014-0664-2

[61] Xenikoudakis G, Ersmark E, Tison JL, Waits L, Kindberg J, Swenson JE, et al. Consequences of a demographic bottleneck on genetic structure and variation in the Scandinavian brown bear. Mol Ecol. 2015;24: 3441–3454. doi: 10.1111/mec.13239 26042479

[62] Sharma SP, Ghazi MG, Katdare S, Dasgupta N, Mondol S, Gupta SK, et al. Microsatellite analysis reveals low genetic diversity in managed populations of the critically endangered gharial (Gavialis gangeticus) in India. Sci Rep. 2021;11: 1–10. doi: 10.1038/s41598-021-85201-w 33707622 PMC7970970

[63] Behling H, Hooghiemstra H. Neotropical Savanna Environments in Space and Time: Late Quaternary Interhemispheric Comparisons. In: Markgraf V, editor. Interhemispheric Climate Linkages. San Diego: Academic Press; 2001. pp. 307–323. doi: 10.1016/B978-012472670-3/50021-5

[64] Wijmstra TA, van der Hammen T. Palynological data on the history of tropical savannas in northern South America. Leidse Geol Meded. 1966;38: 71–83.

[65] Roa P. Estudio de los médanos de los Llanos Centrales de Venezuela: Evidencias de un clima desértico. Acta Biológica Venez. 1979;10: 19–49.

[66] Iriondo M. Climatic changes in the South American plains: Records of a continent-scale oscillation. Quat Int. 1999;57–58: 93–112. doi: 10.1016/S1040-6182(98)00053-6

[67] Martin S. Global diversity of crocodiles (Crocodilia, Reptilia) in freshwater. Hydrobiologia. 2008;595: 587–591. doi: 10.1007/s10750-007-9030-4

[68] Villamarín F, Escobedo-Galván AH, Siroski P, Magnusson WE. Geographic distribution, habitat, reproduction, and conservation status of crocodilians in the Americas. In: Zucoloto RB, Amavet PS, Verdade LM, Farias IP, editors. Conservation Genetics of New World Crocodilians. Cham: Springer International Publishing; 2021. pp. 1–30. doi: 10.1007/978-3-030-56383-7_1

[69] Anzola LF. Abundancia poblacional, aspectos reproductivos y percepción de los habitantes locales, del Caimán LLanero (Crocodylus intermedius, Graves, 1819) en los ríos Lipa, Ele y Cravo Norte del Departamento de Arauca. Boletín la Acad Ciencias Físicas, Matemáticas y Nat. 2017;77: 147–158.

[70] Preciado-Salas BA. Percepción, uso y conservación local del Caimán llanero (Crocodylus intermedius) en el complejo de ríos Cravo Norte, Ele y Lipa (Arauca, Colombia). Pontificia Universidad Javeriana. 2018.

[71] Amavet PS, Barban Zucoloto R, Hrbek T, Farias Pires I. Genetic diversity of new world crocodilians. In: Zucoloto RB, Amavet PS, Verdade LM, Pires IF, editors. Conservation Genetics of New World Crocodilians. Cham: Springer; 2021. pp. 123–152. doi: 10.1007/978-3-030-56383-7

[72] Frankham R, Ballou JD, Eldridge MDB, Lacy RC, Ralls K, Dudash MR, et al. Predicting the probability of outbreeding depression. Conserv Biol. 2011;25: 465–475. doi: 10.1111/j.1523-1739.2011.01662.x 21486369

[73] Weeks AR, Heinze D, Perrin L, Stoklosa J, Hoffmann AA, Van Rooyen A, et al. Genetic rescue increases fitness and aids rapid recovery of an endangered marsupial population. Nat Commun. 2017;8: 1–6. doi: 10.1038/s41467-017-01182-3 29057865 PMC5715156

[74] Bustamante C, editor. El Gran Libro de la Orinoquia Colombiana. Instituto de Investigación de Recursos Biológicos Alexander von Humboldt (IAvH), Deutsche Gessellschaft für Internationale Zusammenarbeit (GIZ) GmbH; 2019.

[75] Jamieson IG, Allendorf FW. How does the 50/500 rule apply to MVPs? Trends Ecol Evol. 2012;27: 578–584. doi: 10.1016/j.tree.2012.07.001 22868005

