# Supplementary material for: First genetic evaluation of a wild population of Crocodylus intermedius: New insights for the recovery of a Critically Endangered species
Source: PLoS One. 2024 Oct 3;19(10):e0311412. doi: 10.1371/journal.pone.0311412 (PMC11449319; doi:10.1371/journal.pone.0311412)
Supplement: S1 Table — (DOCX) [file pone.0311412.s001.docx]

| **Collection number** | **Chip EBTRF** | **Location** | **Approximate coordinates** | **Collection date** |
| --- | --- | --- | --- | --- |
| UNAL:BTBC:10984 \| EBTRF-C-291 | 972752300 | Río Cravo Norte, Cravo Norte, Arauca | 6.530272, -70.816362 | 25/04/2009 |
| UNAL:BTBC:10988 \| EBTRF-C-313 | 972753397 | Río Cravo Norte, Cravo Norte, Arauca | 6.530272, -70.816362 | 25/04/2009 |
| UNAL:BTBC:10990 \| EBTRF-C-321 | 972754014 | Río Cravo Norte, Cravo Norte, Arauca | 6.530272, -70.816362 | 23/04/2009 |
| UNAL:BTBC:10993 \| EBTRF-C-292 | 972759045 | Río Cravo Norte, Cravo Norte, Arauca | 6.530272, -70.816362 | 25/04/2009 |
| UNAL:BTBC:11001 \| EBTRF-C-322 | 972755840 | Río Cravo Norte, Cravo Norte, Arauca | 6.530272, -70.816362 | 23/04/2009 |
| UNAL:BTBC:11002 \| EBTRF-C-312 | 972744397 | Río Cravo Norte, Cravo Norte, Arauca | 6.530272, -70.816362 | 23/04/2009 |
| UNAL:BTBC:11172 \| EBTRF-C-314 | 972759002 | Río Cravo Norte, Cravo Norte, Arauca | 6.530272, -70.816362 | 23/04/2009 |
| UNAL:BTBC:11196 \| EBTRF-C-324 | 972752318 | Río Cravo Norte, Cravo Norte, Arauca | 6.530272, -70.816362 | 25/04/2009 |
| UNAL:BTBC:11323 \| EBTRF-C-320 | 972760517 | Río Cravo Norte, Cravo Norte, Arauca | 6.530272, -70.816362 | 25/04/2009 |
| UNAL:BTBC:12258 | - | Playa Campo Abierto, Río Cravo Norte, Cravo Norte, Arauca | 6.3934861, -70.428436111 | 2016 |
| UNAL:BTBC:12259 | - | Playa Campo Abierto, Río Cravo Norte, Cravo Norte, Arauca | 6.3934861, -70.428436111 | 2016 |
| UNAL:BTBC:12296 | - | Playa Campo Abierto, Río Cravo Norte, Cravo Norte, Arauca | 6.3934861, -70.428436111 | 2016 |
| UNAL:BTBC:12301 | - | Playa Campo Abierto, Río Cravo Norte, Cravo Norte, Arauca | 6.3934861, -70.428436111 | 2016 |
| UNAL:BTBC:12332 | - | Playa Campo Abierto, Río Cravo Norte, Cravo Norte, Arauca | 6.3934861, -70.428436111 | 2016 |
| UNAL:BTBC:12333 | - | Playa Campo Abierto, Río Cravo Norte, Cravo Norte, Arauca | 6.3934861, -70.428436111 | 2016 |
| UNAL:BTBC:12367 | - | Playa Campo Abierto, Río Cravo Norte, Cravo Norte, Arauca | 6.3934861, -70.428436111 | 2016 |
| UNAL:BTBC:12371 | - | Playa Campo Abierto, Río Cravo Norte, Cravo Norte, Arauca | 6.3934861, -70.428436111 | 2016 |
| UNAL:BTBC:13096 | - | Río Ele, Cravo Norte, Arauca | 6.534468, -70.685535 | 10/07/2017 |
| UNAL:BTBC:13098 | - | Río Ele, Cravo Norte, Arauca | 6.532556, -70.674422 | 10/07/2017 |
| UNAL:BTBC:13099 | - | Río Ele, Cravo Norte, Arauca | 6.534733, -70.685381 | 10/07/2017 |
| UNAL:BTBC:13100 | - | Río Ele, Cravo Norte, Arauca | 6.532556, -70.674422 | 10/07/2017 |
| UNAL:BTBC:13101 | - | Río Ele, Cravo Norte, Arauca | 6.534733, -70.685381 | 10/07/2017 |
| UNAL:BTBC:13102 | - | Río Ele, Cravo Norte, Arauca | 6.534733, -70.685381 | 10/07/2017 |
| UNAL:BTBC:13103 | - | Río Ele, Cravo Norte, Arauca | 6.535203, -70.684808 | 10/07/2017 |
| UNAL:BTBC:13104 | - | Río Ele, Cravo Norte, Arauca | 6.535203, -70.684808 | 10/07/2017 |
| UNAL:BTBC:13105 | - | Río Ele, Cravo Norte, Arauca | 6.535203, -70.684808 | 10/07/2017 |
| UNAL:BTBC:13106 | - | Río Ele, Cravo Norte, Arauca | 6.535203, -70.684808 | 10/07/2017 |
| UNAL:BTBC:13107 | - | Río Ele, Cravo Norte, Arauca | 6.535203, -70.684808 | 10/07/2017 |
| UNAL:BTBC:13108 | - | Río Cravo Norte, Cravo Norte, Arauca | 6.390450, -70.432211 | 10/07/2017 |
| UNAL:BTBC:13109 | - | Río Cravo Norte, Cravo Norte, Arauca | 6.390450, -70.432211 | 10/07/2017 |
| UNAL:BTBC:13110 | - | Río Cravo Norte, Cravo Norte, Arauca | 6.390450, -70.432211 | 10/07/2017 |
| UNAL:BTBC:13111 | - | Río Cravo Norte, Cravo Norte, Arauca | 6.390450, -70.432211 | 10/07/2017 |
| UNAL:BTBC:13112 | - | Río Cravo Norte, Cravo Norte, Arauca | 6.390450, -70.432211 | 10/07/2017 |
| UNAL:BTBC:13113 | - | Río Cravo Norte, Cravo Norte, Arauca | 6.390450, -70.432211 | 10/07/2017 |
| UNAL:BTBC:13114 | - | Río Cravo Norte, Cravo Norte, Arauca | 6.390450, -70.432211 | 10/07/2017 |
| UNAL:BTBC:13115 | - | Río Ele, Cravo Norte, Arauca | 6.532556, -70.674422 | 10/07/2017 |
| UNAL:BTBC:13122 | - | Río Ele, Cravo Norte, Arauca | 6.535203, -70.684808 | 10/07/2017 |
| UNAL:BTBC:13123 | - | Río Ele, Cravo Norte, Arauca | 6.534394, -70.685561 | 10/07/2017 |
