# Supplementary material for: First genetic evaluation of a wild population of Crocodylus intermedius: New insights for the recovery of a Critically Endangered species
Source: PLoS One. 2024 Oct 3;19(10):e0311412. doi: 10.1371/journal.pone.0311412 (PMC11449319; doi:10.1371/journal.pone.0311412)
Supplement: S2 Table — (DOCX) [file pone.0311412.s002.docx]

| **Collection number** | **Tissue sample** | **Location** | **Coordinates** | **Collection date** |
| --- | --- | --- | --- | --- |
| UNAL:BTBC:12258 | Caudal scale | Playa Campo Abierto, Río Cravo Norte, Cravo Norte, Arauca | 6.3934861, -70.428436111 | 2016 |
| UNAL:BTBC:12259 | Caudal scale | Playa Campo Abierto, Río Cravo Norte, Cravo Norte, Arauca | 6.3934861, -70.428436111 | 2016 |
| UNAL:BTBC:12262 | Caudal scale | Playa Campo Abierto, Río Cravo Norte, Cravo Norte, Arauca | 6.3934861, -70.428436111 | 2016 |
| UNAL:BTBC:12263 | Caudal scale | Playa Campo Abierto, Río Cravo Norte, Cravo Norte, Arauca | 6.3934861, -70.428436111 | 2016 |
| UNAL:BTBC:12264 | Caudal scale | Playa Campo Abierto, Río Cravo Norte, Cravo Norte, Arauca | 6.3934861, -70.428436111 | 2016 |
| UNAL:BTBC:12265 | Caudal scale | Playa Campo Abierto, Río Cravo Norte, Cravo Norte, Arauca | 6.3934861, -70.428436111 | 2016 |
| UNAL:BTBC:12266 | Caudal scale | Playa Campo Abierto, Río Cravo Norte, Cravo Norte, Arauca | 6.3934861, -70.428436111 | 2016 |
| UNAL:BTBC:12267 | Caudal scale | Playa Campo Abierto, Río Cravo Norte, Cravo Norte, Arauca | 6.3934861, -70.428436111 | 2016 |
| UNAL:BTBC:12268 | Caudal scale | Playa Campo Abierto, Río Cravo Norte, Cravo Norte, Arauca | 6.3934861, -70.428436111 | 2016 |
| UNAL:BTBC:12269 | Caudal scale | Playa Campo Abierto, Río Cravo Norte, Cravo Norte, Arauca | 6.3934861, -70.428436111 | 2016 |
| UNAL:BTBC:12270 | Caudal scale | Playa Campo Abierto, Río Cravo Norte, Cravo Norte, Arauca | 6.3934861, -70.428436111 | 2016 |
| UNAL:BTBC:12271 | Caudal scale | Playa Campo Abierto, Río Cravo Norte, Cravo Norte, Arauca | 6.3934861, -70.428436111 | 2016 |
| UNAL:BTBC:12272 | Caudal scale | Playa Campo Abierto, Río Cravo Norte, Cravo Norte, Arauca | 6.3934861, -70.428436111 | 2016 |
| UNAL:BTBC:12273 | Caudal scale | Playa Campo Abierto, Río Cravo Norte, Cravo Norte, Arauca | 6.3934861, -70.428436111 | 2016 |
| UNAL:BTBC:12274 | Caudal scale | Playa Campo Abierto, Río Cravo Norte, Cravo Norte, Arauca | 6.3934861, -70.428436111 | 2016 |
| UNAL:BTBC:12275 | Caudal scale | Playa Campo Abierto, Río Cravo Norte, Cravo Norte, Arauca | 6.3934861, -70.428436111 | 2016 |
| UNAL:BTBC:12276 | Caudal scale | Playa Campo Abierto, Río Cravo Norte, Cravo Norte, Arauca | 6.3934861, -70.428436111 | 2016 |
| UNAL:BTBC:12277 | Caudal scale | Playa Campo Abierto, Río Cravo Norte, Cravo Norte, Arauca | 6.3934861, -70.428436111 | 2016 |
| UNAL:BTBC:12278 | Caudal scale | Playa Campo Abierto, Río Cravo Norte, Cravo Norte, Arauca | 6.3934861, -70.428436111 | 2016 |
| UNAL:BTBC:12296 | Caudal scale | Playa Campo Abierto, Río Cravo Norte, Cravo Norte, Arauca | 6.3934861, -70.428436111 | 2016 |
| UNAL:BTBC:12297 | Caudal scale | Playa Campo Abierto, Río Cravo Norte, Cravo Norte, Arauca | 6.3934861, -70.428436111 | 2016 |
| UNAL:BTBC:12298 | Caudal scale | Playa Campo Abierto, Río Cravo Norte, Cravo Norte, Arauca | 6.3934861, -70.428436111 | 2016 |
| UNAL:BTBC:12299 | Caudal scale | Playa Campo Abierto, Río Cravo Norte, Cravo Norte, Arauca | 6.3934861, -70.428436111 | 2016 |
| UNAL:BTBC:12300 | Caudal scale | Playa Campo Abierto, Río Cravo Norte, Cravo Norte, Arauca | 6.3934861, -70.428436111 | 2016 |
| UNAL:BTBC:12301 | Caudal scale | Playa Campo Abierto, Río Cravo Norte, Cravo Norte, Arauca | 6.3934861, -70.428436111 | 2016 |
| UNAL:BTBC:12302 | Caudal scale | Playa Campo Abierto, Río Cravo Norte, Cravo Norte, Arauca | 6.3934861, -70.428436111 | 2016 |
| UNAL:BTBC:12303 | Caudal scale | Playa Campo Abierto, Río Cravo Norte, Cravo Norte, Arauca | 6.3934861, -70.428436111 | 2016 |
| UNAL:BTBC:12304 | Caudal scale | Playa Campo Abierto, Río Cravo Norte, Cravo Norte, Arauca | 6.3934861, -70.428436111 | 2016 |
| UNAL:BTBC:12305 | Caudal scale | Playa Campo Abierto, Río Cravo Norte, Cravo Norte, Arauca | 6.3934861, -70.428436111 | 2016 |
| UNAL:BTBC:12306 | Caudal scale | Playa Campo Abierto, Río Cravo Norte, Cravo Norte, Arauca | 6.3934861, -70.428436111 | 2016 |
| UNAL:BTBC:12307 | Caudal scale | Playa Campo Abierto, Río Cravo Norte, Cravo Norte, Arauca | 6.3934861, -70.428436111 | 2016 |
| UNAL:BTBC:12308 | Caudal scale | Playa Campo Abierto, Río Cravo Norte, Cravo Norte, Arauca | 6.3934861, -70.428436111 | 2016 |
| UNAL:BTBC:12309 | Caudal scale | Playa Campo Abierto, Río Cravo Norte, Cravo Norte, Arauca | 6.3934861, -70.428436111 | 2016 |
| UNAL:BTBC:12310 | Caudal scale | Playa Campo Abierto, Río Cravo Norte, Cravo Norte, Arauca | 6.3934861, -70.428436111 | 2016 |
| UNAL:BTBC:12311 | Caudal scale | Playa Campo Abierto, Río Cravo Norte, Cravo Norte, Arauca | 6.3934861, -70.428436111 | 2016 |
| UNAL:BTBC:12312 | Caudal scale | Playa Campo Abierto, Río Cravo Norte, Cravo Norte, Arauca | 6.3934861, -70.428436111 | 2016 |
| UNAL:BTBC:12313 | Caudal scale | Playa Campo Abierto, Río Cravo Norte, Cravo Norte, Arauca | 6.3934861, -70.428436111 | 2016 |
| UNAL:BTBC:12314 | Caudal scale | Playa Campo Abierto, Río Cravo Norte, Cravo Norte, Arauca | 6.3934861, -70.428436111 | 2016 |
| UNAL:BTBC:12315 | Caudal scale | Playa Campo Abierto, Río Cravo Norte, Cravo Norte, Arauca | 6.3934861, -70.428436111 | 2016 |
| UNAL:BTBC:12316 | Caudal scale | Playa Campo Abierto, Río Cravo Norte, Cravo Norte, Arauca | 6.3934861, -70.428436111 | 2016 |
| UNAL:BTBC:12331 | Caudal scale | Playa Campo Abierto, Río Cravo Norte, Cravo Norte, Arauca | 6.3934861, -70.428436111 | 2016 |
| UNAL:BTBC:12332 | Caudal scale | Playa Campo Abierto, Río Cravo Norte, Cravo Norte, Arauca | 6.3934861, -70.428436111 | 2016 |
| UNAL:BTBC:12333 | Caudal scale | Playa Campo Abierto, Río Cravo Norte, Cravo Norte, Arauca | 6.3934861, -70.428436111 | 2016 |
| UNAL:BTBC:12334 | Caudal scale | Playa Campo Abierto, Río Cravo Norte, Cravo Norte, Arauca | 6.3934861, -70.428436111 | 2016 |
| UNAL:BTBC:12335 | Caudal scale | Playa Campo Abierto, Río Cravo Norte, Cravo Norte, Arauca | 6.3934861, -70.428436111 | 2016 |
| UNAL:BTBC:12336 | Caudal scale | Playa Campo Abierto, Río Cravo Norte, Cravo Norte, Arauca | 6.3934861, -70.428436111 | 2016 |
| UNAL:BTBC:12337 | Caudal scale | Playa Campo Abierto, Río Cravo Norte, Cravo Norte, Arauca | 6.3934861, -70.428436111 | 2016 |
| UNAL:BTBC:12338 | Caudal scale | Playa Campo Abierto, Río Cravo Norte, Cravo Norte, Arauca | 6.3934861, -70.428436111 | 2016 |
| UNAL:BTBC:12339 | Caudal scale | Playa Campo Abierto, Río Cravo Norte, Cravo Norte, Arauca | 6.3934861, -70.428436111 | 2016 |
| UNAL:BTBC:12340 | Caudal scale | Playa Campo Abierto, Río Cravo Norte, Cravo Norte, Arauca | 6.3934861, -70.428436111 | 2016 |
| UNAL:BTBC:12341 | Caudal scale | Playa Campo Abierto, Río Cravo Norte, Cravo Norte, Arauca | 6.3934861, -70.428436111 | 2016 |
| UNAL:BTBC:12342 | Caudal scale | Playa Campo Abierto, Río Cravo Norte, Cravo Norte, Arauca | 6.3934861, -70.428436111 | 2016 |
| UNAL:BTBC:12343 | Caudal scale | Playa Campo Abierto, Río Cravo Norte, Cravo Norte, Arauca | 6.3934861, -70.428436111 | 2016 |
| UNAL:BTBC:12344 | Caudal scale | Playa Campo Abierto, Río Cravo Norte, Cravo Norte, Arauca | 6.3934861, -70.428436111 | 2016 |
| UNAL:BTBC:12345 | Caudal scale | Playa Campo Abierto, Río Cravo Norte, Cravo Norte, Arauca | 6.3934861, -70.428436111 | 2016 |
| UNAL:BTBC:12346 | Caudal scale | Playa Campo Abierto, Río Cravo Norte, Cravo Norte, Arauca | 6.3934861, -70.428436111 | 2016 |
| UNAL:BTBC:12347 | Caudal scale | Playa Campo Abierto, Río Cravo Norte, Cravo Norte, Arauca | 6.3934861, -70.428436111 | 2016 |
| UNAL:BTBC:12348 | Caudal scale | Playa Campo Abierto, Río Cravo Norte, Cravo Norte, Arauca | 6.3934861, -70.428436111 | 2016 |
| UNAL:BTBC:12349 | Caudal scale | Playa Campo Abierto, Río Cravo Norte, Cravo Norte, Arauca | 6.3934861, -70.428436111 | 2016 |
| UNAL:BTBC:12350 | Caudal scale | Playa Campo Abierto, Río Cravo Norte, Cravo Norte, Arauca | 6.3934861, -70.428436111 | 2016 |
| UNAL:BTBC:12351 | Caudal scale | Playa Campo Abierto, Río Cravo Norte, Cravo Norte, Arauca | 6.3934861, -70.428436111 | 2016 |
| UNAL:BTBC:12367 | Caudal scale | Playa Campo Abierto, Río Cravo Norte, Cravo Norte, Arauca | 6.3934861, -70.428436111 | 2016 |
| UNAL:BTBC:12368 | Caudal scale | Playa Campo Abierto, Río Cravo Norte, Cravo Norte, Arauca | 6.3934861, -70.428436111 | 2016 |
| UNAL:BTBC:12369 | Caudal scale | Playa Campo Abierto, Río Cravo Norte, Cravo Norte, Arauca | 6.3934861, -70.428436111 | 2016 |
| UNAL:BTBC:12370 | Caudal scale | Playa Campo Abierto, Río Cravo Norte, Cravo Norte, Arauca | 6.3934861, -70.428436111 | 2016 |
| UNAL:BTBC:12371 | Caudal scale | Playa Campo Abierto, Río Cravo Norte, Cravo Norte, Arauca | 6.3934861, -70.428436111 | 2016 |
| UNAL:BTBC:12372 | Caudal scale | Playa Campo Abierto, Río Cravo Norte, Cravo Norte, Arauca | 6.3934861, -70.428436111 | 2016 |
| UNAL:BTBC:12374 | Caudal scale | Playa Campo Abierto, Río Cravo Norte, Cravo Norte, Arauca | 6.3934861, -70.428436111 | 2016 |
| UNAL:BTBC:12375 | Caudal scale | Playa Campo Abierto, Río Cravo Norte, Cravo Norte, Arauca | 6.3934861, -70.428436111 | 2016 |
| UNAL:BTBC:12376 | Caudal scale | Playa Campo Abierto, Río Cravo Norte, Cravo Norte, Arauca | 6.3934861, -70.428436111 | 2016 |
| UNAL:BTBC:12377 | Caudal scale | Playa Campo Abierto, Río Cravo Norte, Cravo Norte, Arauca | 6.3934861, -70.428436111 | 2016 |
| UNAL:BTBC:12378 | Caudal scale | Playa Campo Abierto, Río Cravo Norte, Cravo Norte, Arauca | 6.3934861, -70.428436111 | 2016 |
| UNAL:BTBC:12379 | Caudal scale | Playa Campo Abierto, Río Cravo Norte, Cravo Norte, Arauca | 6.3934861, -70.428436111 | 2016 |
| UNAL:BTBC:12380 | Caudal scale | Playa Campo Abierto, Río Cravo Norte, Cravo Norte, Arauca | 6.3934861, -70.428436111 | 2016 |
| UNAL:BTBC:12381 | Caudal scale | Playa Campo Abierto, Río Cravo Norte, Cravo Norte, Arauca | 6.3934861, -70.428436111 | 2016 |
| UNAL:BTBC:12382 | Caudal scale | Playa Campo Abierto, Río Cravo Norte, Cravo Norte, Arauca | 6.3934861, -70.428436111 | 2016 |
| UNAL:BTBC:12384 | Caudal scale | Playa Campo Abierto, Río Cravo Norte, Cravo Norte, Arauca | 6.3934861, -70.428436111 | 2016 |
| UNAL:BTBC:12385 | Caudal scale | Playa Campo Abierto, Río Cravo Norte, Cravo Norte, Arauca | 6.3934861, -70.428436111 | 2016 |
| UNAL:BTBC:12386 | Caudal scale | Playa Campo Abierto, Río Cravo Norte, Cravo Norte, Arauca | 6.3934861, -70.428436111 | 2016 |
| UNAL:BTBC:12387 | Caudal scale | Playa Campo Abierto, Río Cravo Norte, Cravo Norte, Arauca | 6.3934861, -70.428436111 | 2016 |
| UNAL:BTBC:12388 | Caudal scale | Playa Campo Abierto, Río Cravo Norte, Cravo Norte, Arauca | 6.3934861, -70.428436111 | 2016 |
