# Supplementary material for: First genetic evaluation of a wild population of Crocodylus intermedius: New insights for the recovery of a Critically Endangered species
Source: PLoS One. 2024 Oct 3;19(10):e0311412. doi: 10.1371/journal.pone.0311412 (PMC11449319; doi:10.1371/journal.pone.0311412)
Supplement: S3 Table — (DOCX) [file pone.0311412.s003.docx]

| **Locus** | **Primer sequences (5’-3’)** | **Annealing temperature** | **Multiplex Mix** | **Reference** |
| --- | --- | --- | --- | --- |
| CpP1409 | F: GTTTATGCCCTACTGGTTATCTATC | 57˚C | 1 | Miles and colleagues [1] |
|  | R: CAGTCGGGCGTCATCAGGGAAGGGGATTTAATAAT |  |  |  |
| CpP1610 | F: CAGTCGGGCGTCATCATAGAGGGATTTTGACTGT | 57˚C | 1 | Miles and colleagues [1] |
|  | R: GTTTGATTATTTTGTCTGGGTTCTT |  |  |  |
| CpP302 | F: GTTTGGAACCCAAGAACTTACAAC | 57˚C | 1 | Miles and colleagues [1] |
|  | R: CAGTCGGGCGTCATCATTGGGTTTAGTCAGCACATA |  |  |  |
| CpP305 | F: GTTTGTAGCTGGAACCTGATAGTG | 57˚C | 1 | Miles and colleagues [1] |
|  | R: CAGTCGGGCGTCATCAGGTTAACACGTGGTAACTACA |  |  |  |
| CpP314 | F: GTTTGAAATGCCACTAATACACACA | 57˚C | 1 | Miles and colleagues [1] |
|  | R: CAGTCGGGCGTCATCACCAATTCTTCAGGTCCTTAT |  |  |  |
| CpP3216 | F: CAGTCGGGCGTCATCAGATTAATTCATTGGCTCTC | 57˚C | 1 | Miles and colleagues [1] |
|  | R: GTTTATGCCTTTGCCTTTAG |  |  |  |
| C391 | F: ATGAGTCAGGTGGCAGGTTC | 57˚C | 2 | Fitzsimmons and colleagues [2] |
|  | R: CATAAATACACTTTTGAGCAGCAG |  |  |  |
| CUJ131 | F: GTCCCTTCCAGCCCAAATG | 57˚C | 2 | Fitzsimmons and colleagues [2] |
|  | R: CGTCTGGCCAGAAAACCTGT |  |  |  |
| Cj122 | F: GTTTCATGCTGACTGTTTCTAATCACC | 57˚C | 2 | Fitzsimmons and colleagues [2] |
|  | R: GGAACTACAATTGGTCAACCTCAC |  |  |  |
| Cj16 | F: CATGCAGATTGTTATTCCTGATG | 57˚C | 2 | Fitzsimmons and colleagues [2] |
|  | R: TGTCATGGTGTCAATTAAACTC |  |  |  |
| Cj109 | F: GTATTGTCAACCCCACCGTGTC | 60˚C | 3 | Dever and Densmore [3] |
|  | R: GTTTCCCCTCCACAGATTTACTTGC |  |  |  |
| Cj18 | F: ATCCAAATCCCATGAACCTGAGAG | 60˚C | 3 | Fitzsimmons and colleagues [2] |
|  | R: CCGAGTGCTTACAAGAGGCTGG |  |  |  |
| CU5123 | F: GGGAAGATGACTGGAAT | 60˚C | 3 | Fitzsimmons and colleagues [2] |
|  | R: AAGTGATTAACTAAGCGAGAC |  |  |  |
| Cj101 | F: ACAGGAGGAATGTCGCATAATTG | 57˚C | 4 | Fitzsimmons and colleagues [2] |
|  | R: GTTTATACCGTGCCATCCAAGTTAG |  |  |  |
| Cj127 | F: CCCATAGTTTCCTGTTACCTG | 57˚C | 4 | Fitzsimmons and colleagues [2] |
|  | R: GTTTCCCTCTCTGACTTCAGTGTTG |  |  |  |
| CpDi13 | F: GTTTGTGTCAGCCTATACATGTT | 57˚C | 4 | Miles and colleagues [1] |
|  | R: CAGTCGGGCGTCATCAGTCTCAGAGTATGCCTAGAA |  |  |  |
| CpP801 | F: CAGTCGGGCGTCATCATTGGCATTAGATTGGTAGAC | 57˚C | 4 | Miles and colleagues [1] |
|  | R: GTTTCTATGCCAAAGCTACAAC |  |  |  |

**References**

1. Miles LG, Isberg SR, Moran C, Hagen C, Glenn TC. 253 Novel polymorphic microsatellites for the saltwater crocodile (*Crocodylus porosus*). Conserv Genet. 2009;10(4):963–80.
2. Fitzsimmons NN, Tanksley S, Forstner MRJ, Louis EE, Daglish R, Gratten J, et al. Microsatellite markers for *Crocodylus*: new genetic tools for population genetics, mating system studies and forensics. In: Grigg G, Seebacher F, Franklin CE, editors. Conference on Crocodilian Biology and Evolution. St Lucia Australia: Surrey Beatty & Sons; 2001. p. 51–7.
3. Dever JA, Densmore LD. Microsatellites in Morelet’s Crocodile (*Crocodylus moreletii*) and Their Utility in Addressing Crocodilian Population Genetics Questions. J Herpetol. 2001;35(3):541–4.
