# Supplementary material for: First genetic evaluation of a wild population of Crocodylus intermedius: New insights for the recovery of a Critically Endangered species
Source: PLoS One. 2024 Oct 3;19(10):e0311412. doi: 10.1371/journal.pone.0311412 (PMC11449319; doi:10.1371/journal.pone.0311412)
Supplement: S4 Table — (DOCX) [file pone.0311412.s004.docx]

| **Individual Collection number** | **Allele A** | **Allele B** | **Allele A** | **Allele B** | **Allele A** | **Allele B** | **Allele A** | **Allele B** | **Allele A** | **Allele B** | **Allele A** | **Allele B** | **Allele A** | **Allele B** | **Allele A** | **Allele B** | **Allele A** | **Allele B** | **Allele A** | **Allele B** | **Allele A** | **Allele B** | **Allele A** | **Allele B** | **Allele A** | **Allele B** | **Allele A** | **Allele B** | **Allele A** | **Allele B** | **Allele A** | **Allele B** | **Allele A** | **Allele B** |
| --- | --- | --- | --- | --- | --- | --- | --- | --- | --- | --- | --- | --- | --- | --- | --- | --- | --- | --- | --- | --- | --- | --- | --- | --- | --- | --- | --- | --- | --- | --- | --- | --- | --- | --- |
|  | **CpP3216** | | **CpP305** | | **CpP1409** | | **CpP302** | | **CpP1610** | | **CpP314** | | **Cj16** | | **CU5123** | | **Cj122** | | **Cj18** | | **CUJ131** | | **Cj109** | | **C391** | | **Cj101** | | **CpDi13** | | **Cj127** | | **CpP801** | |
| UNAL:BTBC:10984 \| EBTRF-C-291 | 137 | 137 | 192 | 196 | 249 | 249 | 200 | 200 | 295 | 295 | 254 | 254 | 167 | 167 | 216 | 216 | 378 | 390 | 207 | 211 | 185 | 185 | 384 | 384 | 173 | 179 | 356 | 360 | 360 | 360 | 337 | 337 | 170 | 182 |
| UNAL:BTBC:10988 \| EBTRF-C-313 | 141 | 141 | 176 | 192 | 245 | 249 | 194 | 202 | 295 | 295 | 254 | 254 | 167 | 171 | 216 | 220 | 392 | 392 | 211 | 211 | 185 | 191 | 372 | 384 | 157 | 179 | 356 | 360 | 360 | 362 | 337 | 337 | 178 | 182 |
| UNAL:BTBC:10990 \| EBTRF-C-321 | 141 | 141 | 176 | 192 | 245 | 249 | 194 | 202 | 295 | 295 | 254 | 254 | 167 | 171 | 204 | 220 | 386 | 390 | 211 | 211 | 185 | 191 | 374 | 384 | 153 | 153 | 356 | 360 | 360 | 362 | 337 | 337 | 178 | 182 |
| UNAL:BTBC:10993 \| EBTRF-C-292 | 137 | 137 | 176 | 192 | 249 | 249 | 194 | 194 | 295 | 295 | 262 | 262 | 167 | 167 | 216 | 216 | 380 | 380 | 207 | 211 | 185 | 185 | 384 | 384 | 173 | 173 | 358 | 358 | 360 | 360 | 337 | 337 | 166 | 186 |
| UNAL:BTBC:11001 \| EBTRF-C-322 | 137 | 141 | 176 | 192 | 245 | 249 | 194 | 202 | 295 | 295 | 254 | 254 | 167 | 171 | 216 | 220 | 386 | 390 | 211 | 211 | 185 | 191 | 374 | 384 | 153 | 179 | 356 | 360 | 362 | 362 | 337 | 337 | 178 | 178 |
| UNAL:BTBC:11002 \| EBTRF-C-312 | 141 | 141 | 176 | 192 | 249 | 249 | 194 | 202 | 295 | 295 | 254 | 254 | 141 | 167 | 204 | 220 | 386 | 390 | 207 | 209 | 185 | 191 | 372 | 384 | 153 | 179 | 356 | 360 | 362 | 362 | 337 | 337 | 178 | 178 |
| UNAL:BTBC:11172 \| EBTRF-C-314 | 137 | 141 | 176 | 192 | 245 | 245 | 194 | 194 | 295 | 295 | 254 | 254 | 141 | 171 | 204 | 220 | 386 | 386 | 207 | 209 | 185 | 185 | 372 | 374 | 153 | 157 | 356 | 360 | 360 | 362 | 337 | 337 | 178 | 178 |
| UNAL:BTBC:11196 \| EBTRF-C-324 | 137 | 141 | 176 | 176 | 245 | 249 | 194 | 194 | 295 | 295 | 254 | 258 | 141 | 171 | 204 | 220 | 386 | 386 | 207 | 211 | 185 | 191 | 374 | 374 | 157 | 179 | 356 | 360 | 360 | 362 | 337 | 337 | 178 | 182 |
| UNAL:BTBC:11323 \| EBTRF-C-320 | 137 | 141 | 176 | 192 | 245 | 249 | 194 | 194 | 295 | 295 | 258 | 258 | 141 | 141 | 216 | 220 | 386 | 386 | 209 | 211 | 185 | 191 | 374 | 374 | 153 | 153 | 356 | 360 | 360 | 362 | 337 | 337 | 178 | 182 |
| UNAL:BTBC:12258 | 137 | 137 | 194 | 196 | 245 | 249 | 194 | 194 | 295 | 295 | 254 | 262 | 167 | 173 | 216 | 220 | 380 | 392 | 209 | 211 | 185 | 185 | 372 | 384 | 153 | 173 | 354 | 356 | 360 | 362 | 337 | 337 | 166 | 178 |
| UNAL:BTBC:12259 | 137 | 141 | 194 | 196 | 249 | 249 | 194 | 196 | 295 | 295 | 254 | 262 | 167 | 171 | 216 | 216 | 386 | 390 | 211 | 213 | 185 | 185 | 372 | 384 | 175 | 179 | 356 | 356 | 360 | 362 | 337 | 337 | 178 | 182 |
| UNAL:BTBC:12296 | 137 | 141 | 192 | 196 | 249 | 249 | 194 | 200 | 295 | 295 | 254 | 254 | 167 | 171 | 216 | 216 | 378 | 386 | 207 | 211 | 185 | 193 | 374 | 384 | 161 | 175 | 360 | 360 | 360 | 360 | 337 | 337 | 178 | 178 |
| UNAL:BTBC:12301 | 137 | 137 | 194 | 196 | 245 | 249 | 200 | 202 | 295 | 295 | 254 | 262 | 167 | 167 | 216 | 216 | 378 | 390 | 207 | 213 | 185 | 185 | 384 | 384 | 173 | 175 | 356 | 356 | 360 | 362 | 337 | 337 | 178 | 182 |
| UNAL:BTBC:12332 | 137 | 137 | 196 | 196 | 245 | 249 | 194 | 202 | 295 | 295 | 254 | 262 | 167 | 167 | 216 | 220 | 378 | 380 | 207 | 209 | 185 | 185 | 372 | 384 | 175 | 179 | 356 | 356 | 360 | 360 | 337 | 337 | 166 | 186 |
| UNAL:BTBC:12333 | 137 | 137 | 196 | 196 | 245 | 249 | 194 | 202 | 295 | 295 | 254 | 262 | 167 | 167 | 216 | 220 | 378 | 380 | 207 | 213 | 185 | 185 | 372 | 384 | 153 | 173 | 356 | 356 | 360 | 360 | 337 | 337 | 166 | 178 |
| UNAL:BTBC:12367 | 137 | 141 | 192 | 196 | 249 | 249 | 194 | 196 | 295 | 295 | 254 | 262 | 167 | 167 | 216 | 220 | 380 | 380 | 209 | 213 | 185 | 185 | 372 | 384 | 153 | 175 | 360 | 360 | 360 | 362 | 337 | 337 | 166 | 182 |
| UNAL:BTBC:12371 | 137 | 137 | 192 | 196 | 249 | 249 | 194 | 200 | 295 | 295 | 254 | 262 | 167 | 167 | 216 | 216 | 386 | 390 | 209 | 211 | 185 | 185 | 372 | 384 | 173 | 175 | 360 | 360 | 360 | 360 | 337 | 337 | 166 | 182 |
| UNAL:BTBC:13096 | 137 | 141 | 176 | 192 | 249 | 249 | 194 | 202 | 295 | 295 | 254 | 254 | 167 | 171 | 216 | 216 | 378 | 386 | 207 | 213 | 185 | 185 | 374 | 384 | 175 | 179 | 356 | 360 | 360 | 360 | 337 | 337 | 178 | 186 |
| UNAL:BTBC:13098 | 141 | 141 | 176 | 192 | 245 | 249 | 194 | 200 | 295 | 295 | 254 | 262 | 167 | 171 | 220 | 220 | 386 | 386 | 207 | 211 | 185 | 185 | 372 | 384 | 161 | 175 | 356 | 360 | 360 | 360 | 337 | 337 | 166 | 182 |
| UNAL:BTBC:13099 | 137 | 137 | 176 | 192 | 249 | 249 | 200 | 202 | 295 | 295 | 262 | 262 | 167 | 167 | 216 | 220 | 378 | 386 | 211 | 211 | 185 | 185 | 374 | 384 | 171 | 173 | 356 | 360 | 360 | 362 | 337 | 337 | 178 | 178 |
| UNAL:BTBC:13100 | 141 | 141 | 192 | 196 | 245 | 249 | 196 | 200 | 295 | 295 | 258 | 258 | 167 | 173 | 220 | 220 | 380 | 380 | 209 | 211 | 185 | 185 | 374 | 384 | 161 | 175 | 356 | 360 | 360 | 360 | 337 | 337 | 182 | 182 |
| UNAL:BTBC:13101 | 141 | 141 | 176 | 192 | 249 | 249 | 194 | 200 | 295 | 295 | 254 | 258 | 167 | 171 | 220 | 220 | 386 | 390 | 209 | 211 | 185 | 185 | 372 | 374 | 153 | 171 | 356 | 356 | 362 | 362 | 337 | 337 | 178 | 178 |
| UNAL:BTBC:13102 | 137 | 141 | 176 | 192 | 249 | 249 | 194 | 200 | 295 | 295 | 262 | 262 | 141 | 167 | 216 | 220 | 386 | 386 | 207 | 211 | 185 | 185 | 374 | 374 | 157 | 175 | 356 | 356 | 360 | 362 | 337 | 337 | 178 | 182 |
| UNAL:BTBC:13103 | 141 | 141 | 176 | 192 | 249 | 249 | 194 | 200 | 295 | 295 | 258 | 262 | 141 | 167 | 204 | 216 | 386 | 386 | 207 | 211 | 185 | 185 | 372 | 384 | 157 | 175 | 356 | 356 | 360 | 362 | 337 | 337 | 178 | 182 |
| UNAL:BTBC:13104 | 137 | 137 | 192 | 196 | 249 | 249 | 194 | 200 | 295 | 295 | 262 | 262 | 167 | 167 | 204 | 216 | 378 | 386 | 207 | 213 | 185 | 185 | 372 | 384 | 153 | 175 | 356 | 360 | 360 | 362 | 337 | 337 | 178 | 182 |
| UNAL:BTBC:13105 | 137 | 141 | 176 | 192 | 249 | 249 | 194 | 200 | 295 | 295 | 262 | 262 | 167 | 167 | 216 | 220 | 380 | 390 | 211 | 213 | 185 | 185 | 372 | 384 | 153 | 169 | 356 | 360 | 360 | 362 | 337 | 337 | 182 | 182 |
| UNAL:BTBC:13106 | 137 | 137 | 192 | 196 | 249 | 249 | 194 | 200 | 295 | 295 | 262 | 262 | 167 | 167 | 214 | 220 | 380 | 390 | 209 | 211 | 185 | 185 | 372 | 384 | 153 | 153 | 356 | 360 | 360 | 362 | 337 | 337 | 182 | 182 |
| UNAL:BTBC:13107 | 137 | 141 | 192 | 196 | 249 | 249 | 194 | 200 | 295 | 295 | 254 | 262 | 167 | 171 | 214 | 220 | 390 | 392 | 211 | 213 | 185 | 185 | 372 | 384 | 153 | 153 | 356 | 360 | 362 | 362 | 337 | 337 | 182 | 182 |
| UNAL:BTBC:13108 | 137 | 137 | 196 | 196 | 245 | 249 | 200 | 202 | 295 | 295 | 254 | 262 | 167 | 173 | 216 | 220 | 390 | 392 | 207 | 209 | 185 | 185 | 372 | 372 | 153 | 173 | 356 | 360 | 360 | 360 | 337 | 337 | 178 | 182 |
| UNAL:BTBC:13109 | 137 | 137 | 194 | 196 | 245 | 249 | 194 | 200 | 295 | 295 | 254 | 262 | 167 | 173 | 216 | 216 | 390 | 392 | 207 | 213 | 185 | 185 | 384 | 384 | 173 | 175 | 356 | 360 | 360 | 360 | 337 | 337 | 182 | 186 |
| UNAL:BTBC:13110 | 137 | 141 | 196 | 196 | 249 | 249 | 200 | 200 | 295 | 295 | 262 | 262 | 167 | 167 | 216 | 220 | 390 | 390 | 209 | 213 | 185 | 185 | 372 | 372 | 153 | 175 | 356 | 360 | 358 | 362 | 337 | 337 | 182 | 182 |
| UNAL:BTBC:13111 | 137 | 141 | 196 | 196 | 249 | 249 | 194 | 200 | 295 | 295 | 254 | 262 | 167 | 167 | 216 | 216 | 380 | 390 | 209 | 209 | 185 | 185 | 384 | 384 | 175 | 175 | 360 | 360 | 360 | 362 | 337 | 337 | 182 | 182 |
| UNAL:BTBC:13112 | 137 | 141 | 176 | 192 | 249 | 249 | 194 | 200 | 295 | 295 | 254 | 262 | 167 | 167 | 204 | 216 | 386 | 390 | 211 | 213 | 185 | 185 | 372 | 384 | 173 | 175 | 360 | 360 | 360 | 360 | 337 | 337 | 178 | 186 |
| UNAL:BTBC:13113 | 137 | 141 | 176 | 196 | 249 | 249 | 194 | 194 | 295 | 295 | 254 | 262 | 167 | 167 | 216 | 216 | 380 | 380 | 209 | 209 | 185 | 185 | 372 | 372 | 153 | 175 | 356 | 360 | 360 | 362 | 337 | 337 | 166 | 182 |
| UNAL:BTBC:13114 | 141 | 141 | 176 | 196 | 249 | 249 | 194 | 202 | 295 | 295 | 254 | 262 | 167 | 167 | 216 | 216 | 378 | 386 | 207 | 213 | 185 | 193 | 374 | 384 | 179 | 179 | 356 | 360 | 360 | 362 | 337 | 337 | 178 | 178 |
| UNAL:BTBC:13115 | 137 | 137 | 196 | 196 | 245 | 249 | 194 | 194 | 295 | 295 | 254 | 262 | 167 | 173 | 216 | 220 | 380 | 392 | 209 | 211 | 185 | 185 | 384 | 384 | 153 | 173 | 356 | 360 | 360 | 362 | 337 | 337 | 166 | 166 |
| UNAL:BTBC:13122 | 137 | 141 | 176 | 176 | 249 | 249 | 194 | 200 | 295 | 295 | 254 | 262 | 167 | 167 | 204 | 216 | 378 | 386 | 209 | 211 | 185 | 185 | 372 | 384 | 153 | 175 | 356 | 360 | 360 | 360 | 337 | 337 | 166 | 178 |
| UNAL:BTBC:13123 | 141 | 141 | 176 | 192 | 249 | 249 | 194 | 202 | 295 | 295 | 254 | 262 | 167 | 171 | 216 | 220 | 378 | 386 | 211 | 213 | 185 | 185 | 374 | 384 | 173 | 175 | 356 | 360 | 360 | 360 | 337 | 337 | 178 | 178 |
