# Supplementary material for: First genetic evaluation of a wild population of Crocodylus intermedius: New insights for the recovery of a Critically Endangered species
Source: PLoS One. 2024 Oct 3;19(10):e0311412. doi: 10.1371/journal.pone.0311412 (PMC11449319; doi:10.1371/journal.pone.0311412)
Supplement: S5 Table — (DOCX) [file pone.0311412.s005.docx]

| **Individual Collection number** | **Allele A** | **Allele B** | **Allele A** | **Allele B** | **Allele A** | **Allele B** | **Allele A** | **Allele B** | **Allele A** | **Allele B** | **Allele A** | **Allele B** | **Allele A** | **Allele B** | **Allele A** | **Allele B** | **Allele A** | **Allele B** | **Allele A** | **Allele B** | **Allele A** | **Allele B** | **Allele A** | **Allele B** | **Allele A** | **Allele B** | **Allele A** | **Allele B** | **Allele A** | **Allele B** | **Allele A** | **Allele B** | **Allele A** | **Allele B** |
| --- | --- | --- | --- | --- | --- | --- | --- | --- | --- | --- | --- | --- | --- | --- | --- | --- | --- | --- | --- | --- | --- | --- | --- | --- | --- | --- | --- | --- | --- | --- | --- | --- | --- | --- |
|  | **CpP3216** | | **CpP305** | | **CpP1409** | | **CpP302** | | **CpP1610** | | **CpP314** | | **Cj16** | | **CU5123** | | **Cj122** | | **Cj18** | | **CUJ131** | | **Cj109** | | **C391** | | **Cj101** | | **CpDi13** | | **Cj127** | | **CpP801** | |
| UNAL:BTBC:12258 | 137 | 137 | 194 | 196 | 245 | 249 | 194 | 194 | 295 | 295 | 254 | 262 | 167 | 173 | 216 | 220 | 380 | 392 | 209 | 211 | 185 | 185 | 372 | 384 | 153 | 173 | 354 | 356 | 360 | 362 | 337 | 337 | 166 | 178 |
| UNAL:BTBC:12259 | 137 | 141 | 194 | 196 | 249 | 249 | 194 | 196 | 295 | 295 | 254 | 262 | 167 | 171 | 216 | 216 | 386 | 390 | 211 | 213 | 185 | 185 | 372 | 384 | 175 | 179 | 356 | 356 | 360 | 362 | 337 | 337 | 178 | 182 |
| UNAL:BTBC:12262 | 137 | 137 | 196 | 196 | 249 | 249 | 200 | 202 | 295 | 295 | 254 | 262 | 167 | 167 | 216 | 216 | 378 | 390 | 207 | 209 | 185 | 185 | 384 | 384 | 173 | 175 | 356 | 360 | 360 | 362 | 337 | 337 | 178 | 182 |
| UNAL:BTBC:12263 | 137 | 137 | 176 | 192 | 249 | 249 | 194 | 200 | 295 | 295 | 254 | 254 | 167 | 167 | 204 | 216 | 386 | 390 | 207 | 213 | 185 | 193 | 384 | 384 | 161 | 175 | 356 | 360 | 360 | 360 | 337 | 337 | 166 | 178 |
| UNAL:BTBC:12264 | 137 | 141 | 192 | 192 | 249 | 249 | 194 | 194 | 295 | 295 | 258 | 262 | 167 | 171 | 216 | 220 | 386 | 386 | 211 | 211 | 185 | 185 | 372 | 372 | 157 | 179 | 356 | 360 | 360 | 362 | 337 | 337 | 182 | 186 |
| UNAL:BTBC:12265 | 137 | 141 | 176 | 192 | 249 | 249 | 194 | 194 | 295 | 295 | 258 | 262 | 141 | 167 | 216 | 220 | 386 | 386 | 211 | 211 | 185 | 191 | 372 | 374 | 153 | 179 | 356 | 356 | 362 | 362 | 337 | 337 | 182 | 186 |
| UNAL:BTBC:12266 | 137 | 137 | 194 | 196 | 249 | 249 | 194 | 194 | 295 | 295 | 254 | 262 | 167 | 167 | 216 | 220 | 378 | 380 | 209 | 211 | 185 | 185 | 372 | 372 | 153 | 173 | 360 | 360 | 360 | 360 | 337 | 337 | 166 | 178 |
| UNAL:BTBC:12267 | 137 | 141 | 176 | 196 | 249 | 249 | 194 | 200 | 295 | 295 | 254 | 262 | 167 | 167 | 216 | 216 | 380 | 390 | 207 | 209 | 185 | 185 | 372 | 372 | 153 | 175 | 360 | 360 | 360 | 360 | 337 | 337 | 182 | 182 |
| UNAL:BTBC:12268 | 137 | 141 | 176 | 196 | 249 | 249 | 194 | 194 | 295 | 295 | 254 | 262 | 167 | 167 | 216 | 220 | 380 | 380 | 209 | 213 | 185 | 185 | 372 | 384 | 173 | 175 | 360 | 360 | 362 | 362 | 337 | 337 | 166 | 166 |
| UNAL:BTBC:12269 | 137 | 141 | 192 | 196 | 249 | 249 | 194 | 202 | 295 | 295 | 254 | 254 | 167 | 171 | 204 | 216 | 378 | 386 | 211 | 213 | 193 | 193 | 384 | 384 | 161 | 179 | 356 | 356 | 360 | 362 | 337 | 337 | 178 | 178 |
| UNAL:BTBC:12270 | 137 | 141 | 176 | 192 | 249 | 249 | 202 | 202 | 295 | 295 | 254 | 254 | 167 | 167 | 216 | 216 | 378 | 378 | 211 | 213 | 185 | 185 | 384 | 384 | 173 | 173 | 360 | 360 | 360 | 362 | 337 | 337 | 178 | 182 |
| UNAL:BTBC:12271 | 137 | 141 | 192 | 192 | 245 | 249 | 194 | 194 | 295 | 295 | 258 | 262 | 141 | 167 | 220 | 220 | 386 | 386 | 209 | 211 | 191 | 193 | 374 | 384 | 153 | 171 | 356 | 360 | 362 | 362 | 337 | 337 | 178 | 186 |
| UNAL:BTBC:12272 | 137 | 137 | 192 | 196 | 249 | 249 | 194 | 200 | 295 | 295 | 254 | 262 | 167 | 171 | 216 | 220 | 386 | 390 | 209 | 211 | 185 | 185 | 374 | 384 | 153 | 179 | 356 | 356 | 360 | 362 | 337 | 337 | 178 | 182 |
| UNAL:BTBC:12273 | 137 | 137 | 192 | 192 | 249 | 249 | 194 | 196 | 295 | 295 | 262 | 262 | 167 | 167 | 204 | 216 | 386 | 390 | 211 | 211 | 185 | 185 | 372 | 384 | 153 | 173 | 360 | 360 | 360 | 360 | 337 | 337 | 182 | 182 |
| UNAL:BTBC:12274 | 137 | 137 | 176 | 192 | 245 | 249 | 194 | 194 | 295 | 295 | 258 | 262 | 141 | 167 | 216 | 220 | 386 | 386 | 211 | 211 | 191 | 193 | 372 | 374 | 153 | 179 | 356 | 360 | 362 | 362 | 337 | 337 | 182 | 182 |
| UNAL:BTBC:12275 | 137 | 137 | 196 | 196 | 249 | 249 | 200 | 200 | 295 | 295 | 254 | 262 | 167 | 167 | 216 | 216 | 390 | 390 | 211 | 213 | 185 | 193 | 372 | 372 | 175 | 179 | 356 | 360 | 360 | 360 | 337 | 337 | 178 | 182 |
| UNAL:BTBC:12276 | 137 | 141 | 176 | 196 | 249 | 249 | 194 | 200 | 295 | 295 | 254 | 254 | 167 | 171 | 216 | 216 | 386 | 390 | 211 | 211 | 193 | 193 | 374 | 384 | 161 | 179 | 360 | 360 | 360 | 360 | 337 | 337 | 166 | 178 |
| UNAL:BTBC:12277 | 137 | 137 | 192 | 192 | 249 | 249 | 194 | 194 | 295 | 295 | 258 | 258 | 141 | 171 | 220 | 220 | 386 | 386 | 211 | 211 | 185 | 185 | 372 | 384 | 153 | 179 | 356 | 360 | 362 | 362 | 337 | 337 | 182 | 182 |
| UNAL:BTBC:12278 | 137 | 137 | 192 | 196 | 249 | 249 | 194 | 202 | 295 | 295 | 254 | 262 | 167 | 167 | 204 | 216 | 378 | 386 | 209 | 211 | 185 | 185 | 372 | 384 | 173 | 179 | 358 | 360 | 360 | 362 | 337 | 337 | 166 | 178 |
| UNAL:BTBC:12296 | 137 | 141 | 192 | 196 | 249 | 249 | 194 | 200 | 295 | 295 | 254 | 254 | 167 | 171 | 216 | 216 | 378 | 386 | 207 | 211 | 185 | 193 | 374 | 384 | 161 | 175 | 360 | 360 | 360 | 360 | 337 | 337 | 178 | 178 |
| UNAL:BTBC:12297 | 137 | 141 | 176 | 196 | 249 | 249 | 194 | 202 | 295 | 295 | 254 | 254 | 167 | 171 | 216 | 216 | 378 | 386 | 211 | 213 | 193 | 193 | 374 | 384 | 179 | 179 | 356 | 360 | 360 | 360 | 337 | 337 | 178 | 178 |
| UNAL:BTBC:12298 | 137 | 141 | 192 | 192 | 245 | 249 | 194 | 194 | 295 | 295 | 254 | 258 | 141 | 171 | 216 | 220 | 386 | 386 | 209 | 211 | 185 | 191 | 374 | 374 | 157 | 179 | 356 | 360 | 360 | 362 | 337 | 337 | 178 | 186 |
| UNAL:BTBC:12299 | 137 | 141 | 176 | 196 | 249 | 249 | 194 | 200 | 295 | 295 | 254 | 254 | 167 | 171 | 216 | 216 | 386 | 390 | 211 | 213 | 185 | 193 | 384 | 384 | 161 | 175 | 356 | 360 | 360 | 362 | 337 | 337 | 166 | 178 |
| UNAL:BTBC:12300 | 137 | 141 | 176 | 196 | 249 | 249 | 194 | 200 | 295 | 295 | 254 | 254 | 167 | 171 | 216 | 216 | 386 | 390 | 207 | 211 | 185 | 193 | 374 | 384 | 175 | 179 | 354 | 356 | 360 | 360 | 337 | 337 | 166 | 178 |
| UNAL:BTBC:12301 | 137 | 137 | 194 | 196 | 245 | 249 | 200 | 202 | 295 | 295 | 254 | 262 | 167 | 167 | 216 | 216 | 378 | 390 | 207 | 213 | 185 | 185 | 384 | 384 | 173 | 175 | 356 | 356 | 360 | 362 | 337 | 337 | 178 | 182 |
| UNAL:BTBC:12302 | 137 | 141 | 176 | 196 | 249 | 249 | 200 | 200 | 295 | 295 | 254 | 262 | 167 | 167 | 216 | 220 | 380 | 390 | 209 | 213 | 185 | 185 | 372 | 372 | 173 | 175 | 360 | 360 | 360 | 360 | 337 | 337 | 166 | 182 |
| UNAL:BTBC:12303 | 137 | 137 | 196 | 196 | 249 | 249 | 200 | 202 | 295 | 295 | 254 | 262 | 167 | 167 | 216 | 220 | 378 | 390 | 207 | 213 | 185 | 185 | 372 | 384 | 153 | 173 | 356 | 360 | 360 | 362 | 337 | 337 | 182 | 186 |
| UNAL:BTBC:12304 | 137 | 137 | 196 | 196 | 245 | 249 | 200 | 202 | 295 | 295 | 254 | 262 | 167 | 167 | 216 | 220 | 378 | 390 | 211 | 213 | 185 | 185 | 372 | 372 | 153 | 173 | 356 | 360 | 360 | 362 | 337 | 337 | 178 | 182 |
| UNAL:BTBC:12305 | 137 | 137 | 192 | 192 | 249 | 249 | 194 | 196 | 295 | 295 | 258 | 262 | 167 | 167 | 204 | 216 | 386 | 390 | 209 | 211 | 185 | 185 | 384 | 384 | 173 | 179 | 360 | 360 | 360 | 362 | 337 | 337 | 166 | 182 |
| UNAL:BTBC:12306 | 137 | 141 | 196 | 196 | 249 | 249 | 194 | 200 | 295 | 295 | 254 | 262 | 167 | 167 | 216 | 216 | 380 | 390 | 209 | 209 | 185 | 185 | 384 | 384 | 173 | 175 | 358 | 360 | 360 | 360 | 337 | 337 | 182 | 182 |
| UNAL:BTBC:12307 | 137 | 141 | 176 | 196 | 249 | 249 | 194 | 200 | 295 | 295 | 254 | 254 | 167 | 171 | 204 | 216 | 386 | 390 | 207 | 213 | 193 | 193 | 384 | 384 | 175 | 179 | 360 | 360 | 360 | 360 | 337 | 337 | 166 | 178 |
| UNAL:BTBC:12308 | 137 | 141 | 196 | 196 | 249 | 249 | 194 | 200 | 295 | 295 | 262 | 262 | 167 | 167 | 216 | 216 | 390 | 390 | 207 | 213 | 185 | 185 | 372 | 384 | 153 | 175 | 356 | 360 | 360 | 360 | 337 | 337 | 166 | 182 |
| UNAL:BTBC:12309 | 137 | 141 | 176 | 196 | 249 | 249 | 194 | 200 | 295 | 295 | 254 | 262 | 167 | 167 | 220 | 220 | 380 | 380 | 209 | 209 | 185 | 185 | 372 | 384 | 153 | 153 | 360 | 360 | 360 | 360 | 337 | 337 | 182 | 182 |
| UNAL:BTBC:12310 | 137 | 137 | 194 | 196 | 245 | 249 | 194 | 194 | 295 | 295 | 254 | 262 | 167 | 167 | 216 | 216 | 378 | 380 | 207 | 209 | 185 | 185 | 372 | 372 | 175 | 179 | 356 | 360 | 360 | 362 | 337 | 337 | 166 | 178 |
| UNAL:BTBC:12311 | 137 | 141 | 192 | 192 | 249 | 249 | 194 | 194 | 295 | 295 | 254 | 258 | 141 | 141 | 216 | 220 | 386 | 386 | 209 | 211 | 191 | 193 | 374 | 374 | 153 | 179 | 356 | 356 | 362 | 362 | 337 | 337 | 178 | 186 |
| UNAL:BTBC:12312 | 137 | 137 | 194 | 196 | 245 | 249 | 194 | 194 | 295 | 295 | 254 | 262 | 167 | 167 | 220 | 220 | 380 | 380 | 209 | 209 | 185 | 185 | 372 | 384 | 153 | 153 | 360 | 360 | 360 | 360 | 337 | 337 | 182 | 182 |
| UNAL:BTBC:12313 | 137 | 137 | 196 | 196 | 245 | 249 | 194 | 200 | 295 | 295 | 254 | 262 | 167 | 167 | 216 | 220 | 380 | 390 | 209 | 211 | 185 | 185 | 372 | 384 | 153 | 179 | 356 | 360 | 360 | 362 | 337 | 337 | 166 | 178 |
| UNAL:BTBC:12314 | 137 | 141 | 196 | 196 | 249 | 249 | 194 | 200 | 295 | 295 | 262 | 262 | 167 | 167 | 216 | 220 | 378 | 380 | 209 | 211 | 185 | 185 | 384 | 384 | 153 | 173 | 354 | 356 | 360 | 360 | 337 | 337 | 166 | 186 |
| UNAL:BTBC:12315 | 137 | 141 | 176 | 196 | 249 | 249 | 194 | 200 | 295 | 295 | 254 | 262 | 167 | 173 | 216 | 220 | 380 | 392 | 211 | 213 | 185 | 185 | 372 | 384 | 153 | 179 | 356 | 356 | 360 | 362 | 337 | 337 | 166 | 178 |
| UNAL:BTBC:12316 | 137 | 141 | 192 | 192 | 249 | 249 | 194 | 194 | 295 | 295 | 258 | 258 | 141 | 171 | 220 | 220 | 386 | 386 | 211 | 211 | 191 | 193 | 374 | 374 | 157 | 171 | 356 | 356 | 362 | 362 | 337 | 337 | 182 | 186 |
| UNAL:BTBC:12331 | 137 | 141 | 192 | 196 | 249 | 249 | 194 | 200 | 295 | 295 | 254 | 254 | 167 | 171 | 216 | 216 | 386 | 390 | 211 | 213 | 193 | 193 | 384 | 384 | 161 | 179 | 354 | 356 | 360 | 362 | 337 | 337 | 166 | 178 |
| UNAL:BTBC:12332 | 137 | 137 | 196 | 196 | 245 | 249 | 194 | 202 | 295 | 295 | 254 | 262 | 167 | 167 | 216 | 220 | 378 | 380 | 207 | 209 | 185 | 185 | 372 | 384 | 175 | 179 | 356 | 356 | 360 | 360 | 337 | 337 | 166 | 186 |
| UNAL:BTBC:12333 | 137 | 137 | 196 | 196 | 245 | 249 | 194 | 202 | 295 | 295 | 254 | 262 | 167 | 167 | 216 | 220 | 378 | 380 | 207 | 213 | 185 | 185 | 372 | 384 | 153 | 173 | 356 | 356 | 360 | 360 | 337 | 337 | 166 | 178 |
| UNAL:BTBC:12334 | 137 | 141 | 176 | 192 | 249 | 249 | 194 | 200 | 295 | 295 | 254 | 262 | 167 | 167 | 216 | 216 | 378 | 386 | 211 | 213 | 185 | 185 | 372 | 384 | 175 | 179 | 360 | 360 | 362 | 362 | 337 | 337 | 178 | 186 |
| UNAL:BTBC:12335 | 137 | 141 | 196 | 196 | 249 | 249 | 194 | 200 | 295 | 295 | 254 | 254 | 167 | 171 | 204 | 216 | 386 | 390 | 207 | 211 | 185 | 193 | 384 | 384 | 179 | 179 | 356 | 356 | 360 | 360 | 337 | 337 | 166 | 178 |
| UNAL:BTBC:12336 | 137 | 137 | 196 | 196 | 245 | 249 | 194 | 200 | 295 | 295 | 254 | 262 | 167 | 173 | 216 | 220 | 390 | 392 | 209 | 211 | 185 | 185 | 384 | 384 | 153 | 179 | 356 | 360 | 360 | 360 | 337 | 337 | 166 | 178 |
| UNAL:BTBC:12337 | 137 | 141 | 192 | 192 | 249 | 249 | 194 | 194 | 295 | 295 | 258 | 262 | 167 | 171 | 220 | 220 | 386 | 386 | 211 | 211 | 185 | 193 | 374 | 374 | 153 | 171 | 356 | 356 | 362 | 362 | 337 | 337 | 178 | 186 |
| UNAL:BTBC:12338 | 137 | 137 | 194 | 196 | 245 | 249 | 194 | 202 | 295 | 295 | 254 | 262 | 167 | 167 | 216 | 216 | 380 | 392 | 207 | 209 | 185 | 185 | 384 | 384 | 175 | 179 | 356 | 360 | 360 | 362 | 337 | 337 | 166 | 186 |
| UNAL:BTBC:12339 | 137 | 141 | 192 | 192 | 245 | 249 | 194 | 194 | 295 | 295 | 258 | 258 | 141 | 141 | 216 | 220 | 386 | 386 | 209 | 211 | 185 | 193 | 374 | 374 | 157 | 179 | 356 | 356 | 362 | 362 | 337 | 337 | 178 | 186 |
| UNAL:BTBC:12340 | 137 | 137 | 194 | 196 | 249 | 249 | 194 | 194 | 295 | 295 | 254 | 262 | 167 | 173 | 216 | 220 | 380 | 392 | 207 | 213 | 185 | 185 | 372 | 384 | 153 | 173 | 360 | 360 | 360 | 360 | 337 | 337 | 166 | 186 |
| UNAL:BTBC:12341 | 137 | 141 | 176 | 196 | 249 | 249 | 194 | 200 | 295 | 295 | 254 | 254 | 167 | 171 | 204 | 216 | 386 | 390 | 211 | 213 | 185 | 193 | 384 | 384 | 179 | 179 | 360 | 360 | 360 | 360 | 337 | 337 | 166 | 178 |
| UNAL:BTBC:12342 | 137 | 141 | 176 | 192 | 249 | 249 | 202 | 202 | 295 | 295 | 254 | 254 | 167 | 167 | 204 | 216 | 378 | 378 | 209 | 211 | 185 | 185 | 372 | 384 | 173 | 175 | 360 | 360 | 360 | 362 | 337 | 337 | 178 | 186 |
| UNAL:BTBC:12343 | 137 | 137 | 196 | 196 | 249 | 249 | 194 | 200 | 295 | 295 | 254 | 262 | 167 | 171 | 204 | 216 | 386 | 390 | 209 | 211 | 185 | 193 | 372 | 372 | 175 | 179 | 356 | 360 | 360 | 362 | 337 | 337 | 182 | 186 |
| UNAL:BTBC:12344 | 137 | 141 | 176 | 192 | 245 | 249 | 194 | 194 | 295 | 295 | 254 | 258 | 141 | 171 | 220 | 220 | 386 | 386 | 209 | 211 | 191 | 191 | 374 | 374 | 153 | 171 | 356 | 356 | 362 | 362 | 337 | 337 | 182 | 186 |
| UNAL:BTBC:12345 | 137 | 137 | 194 | 196 | 245 | 249 | 194 | 194 | 295 | 295 | 254 | 262 | 167 | 173 | 216 | 216 | 380 | 392 | 211 | 213 | 185 | 185 | 372 | 372 | 175 | 179 | 356 | 356 | 360 | 362 | 337 | 337 | 166 | 186 |
| UNAL:BTBC:12346 | 137 | 137 | 192 | 192 | 249 | 249 | 194 | 196 | 295 | 295 | 258 | 262 | 167 | 171 | 216 | 216 | 378 | 386 | 211 | 213 | 193 | 193 | 374 | 384 | 179 | 179 | 356 | 360 | 360 | 362 | 337 | 337 | 178 | 178 |
| UNAL:BTBC:12347 | 137 | 141 | 192 | 196 | 249 | 249 | 194 | 200 | 295 | 295 | 254 | 262 | 167 | 171 | 204 | 216 | 386 | 390 | 207 | 211 | 185 | 193 | 374 | 384 | 175 | 179 | 356 | 356 | 360 | 360 | 337 | 337 | 166 | 178 |
| UNAL:BTBC:12348 | 137 | 141 | 192 | 196 | 249 | 249 | 194 | 194 | 295 | 295 | 258 | 262 | 141 | 167 | 220 | 220 | 386 | 386 | 209 | 211 | 185 | 193 | 374 | 384 | 157 | 171 | 356 | 360 | 360 | 362 | 337 | 337 | 182 | 186 |
| UNAL:BTBC:12349 | 137 | 137 | 176 | 196 | 249 | 249 | 194 | 202 | 295 | 295 | 254 | 254 | 167 | 171 | 216 | 216 | 378 | 386 | 207 | 213 | 185 | 193 | 374 | 384 | 161 | 179 | 356 | 360 | 360 | 360 | 337 | 337 | 178 | 178 |
| UNAL:BTBC:12350 | 137 | 137 | 196 | 196 | 245 | 249 | 200 | 202 | 295 | 295 | 254 | 262 | 167 | 173 | 216 | 220 | 390 | 392 | 207 | 209 | 185 | 185 | 372 | 372 | 153 | 173 | 356 | 360 | 360 | 360 | 337 | 337 | 178 | 182 |
| UNAL:BTBC:12351 | 137 | 137 | 192 | 196 | 249 | 249 | 200 | 200 | 295 | 295 | 262 | 262 | 167 | 167 | 204 | 216 | 390 | 390 | 211 | 213 | 185 | 193 | 372 | 374 | 153 | 161 | 356 | 360 | 360 | 362 | 337 | 337 | 182 | 186 |
| UNAL:BTBC:12367 | 137 | 141 | 192 | 196 | 249 | 249 | 194 | 196 | 295 | 295 | 254 | 262 | 167 | 167 | 216 | 220 | 380 | 380 | 209 | 213 | 185 | 185 | 372 | 384 | 153 | 175 | 360 | 360 | 360 | 362 | 337 | 337 | 166 | 182 |
| UNAL:BTBC:12368 | 137 | 137 | 192 | 192 | 249 | 249 | 194 | 196 | 295 | 295 | 258 | 262 | 167 | 167 | 204 | 220 | 378 | 378 | 209 | 211 | 185 | 185 | 372 | 382 | 153 | 173 | 360 | 360 | 360 | 360 | 337 | 337 | 182 | 182 |
| UNAL:BTBC:12369 | 137 | 141 | 192 | 192 | 245 | 249 | 194 | 194 | 295 | 295 | 258 | 262 | 141 | 167 | 216 | 220 | 386 | 386 | 209 | 211 | 185 | 185 | 372 | 374 | 153 | 179 | 354 | 356 | 362 | 362 | 337 | 337 | 182 | 186 |
| UNAL:BTBC:12370 | 137 | 137 | 192 | 192 | 249 | 249 | 194 | 196 | 295 | 295 | 258 | 262 | 141 | 171 | 216 | 220 | 386 | 386 | 211 | 211 | 185 | 191 | 372 | 384 | 153 | 171 | 356 | 356 | 360 | 362 | 337 | 337 | 182 | 186 |
| UNAL:BTBC:12371 | 137 | 137 | 192 | 196 | 249 | 249 | 194 | 200 | 295 | 295 | 254 | 262 | 167 | 167 | 216 | 216 | 386 | 390 | 209 | 211 | 185 | 185 | 372 | 384 | 173 | 175 | 360 | 360 | 360 | 360 | 337 | 337 | 166 | 182 |
| UNAL:BTBC:12372 | 137 | 137 | 192 | 192 | 249 | 249 | 194 | 196 | 295 | 295 | 258 | 262 | 167 | 171 | 216 | 216 | 378 | 386 | 207 | 211 | 185 | 193 | 372 | 384 | 153 | 179 | 356 | 360 | 360 | 362 | 337 | 337 | 182 | 182 |
| UNAL:BTBC:12374 | 137 | 141 | 196 | 196 | 249 | 249 | 194 | 200 | 295 | 295 | 254 | 262 | 167 | 171 | 220 | 220 | 386 | 390 | 209 | 209 | 185 | 191 | 372 | 372 | 153 | 175 | 356 | 360 | 362 | 362 | 337 | 337 | 178 | 182 |
| UNAL:BTBC:12375 | 141 | 141 | 176 | 196 | 249 | 249 | 194 | 200 | 295 | 295 | 254 | 262 | 167 | 167 | 204 | 216 | 386 | 390 | 207 | 213 | 185 | 193 | 374 | 384 | 179 | 179 | 356 | 360 | 360 | 360 | 337 | 337 | 166 | 178 |
| UNAL:BTBC:12376 | 137 | 137 | 176 | 192 | 249 | 249 | 194 | 200 | 295 | 295 | 254 | 262 | 167 | 171 | 204 | 216 | 386 | 390 | 207 | 213 | 185 | 193 | 374 | 384 | 161 | 179 | 356 | 356 | 360 | 362 | 337 | 337 | 178 | 186 |
| UNAL:BTBC:12377 | 137 | 137 | 192 | 196 | 249 | 249 | 194 | 200 | 295 | 295 | 262 | 262 | 167 | 167 | 204 | 220 | 380 | 390 | 209 | 211 | 185 | 185 | 372 | 384 | 153 | 161 | 356 | 360 | 360 | 360 | 337 | 337 | 166 | 178 |
| UNAL:BTBC:12378 | 137 | 137 | 192 | 192 | 249 | 249 | 194 | 196 | 295 | 295 | 258 | 262 | 167 | 167 | 216 | 220 | 378 | 380 | 209 | 211 | 185 | 185 | 372 | 384 | 153 | 173 | 356 | 360 | 360 | 360 | 337 | 337 | 186 | 186 |
| UNAL:BTBC:12379 | 137 | 137 | 192 | 192 | 249 | 249 | 194 | 194 | 295 | 295 | 254 | 262 | 167 | 171 | 204 | 216 | 380 | 386 | 211 | 213 | 185 | 193 | 372 | 372 | 153 | 161 | 360 | 360 | 360 | 360 | 337 | 337 | 182 | 182 |
| UNAL:BTBC:12380 | 137 | 137 | 192 | 196 | 249 | 249 | 194 | 194 | 295 | 295 | 254 | 262 | 167 | 171 | 220 | 220 | 386 | 386 | 207 | 211 | 185 | 185 | 372 | 374 | 153 | 171 | 356 | 356 | 360 | 362 | 337 | 337 | 182 | 186 |
| UNAL:BTBC:12381 | 137 | 137 | 176 | 192 | 249 | 249 | 194 | 200 | 295 | 295 | 254 | 262 | 167 | 167 | 204 | 216 | 386 | 390 | 207 | 211 | 185 | 193 | 384 | 384 | 179 | 179 | 356 | 360 | 360 | 360 | 337 | 337 | 166 | 178 |
| UNAL:BTBC:12382 | 137 | 137 | 192 | 192 | 249 | 249 | 194 | 196 | 295 | 295 | 258 | 262 | 141 | 167 | 220 | 220 | 386 | 386 | 211 | 211 | 185 | 191 | 372 | 374 | 153 | 171 | 356 | 360 | 362 | 362 | 337 | 337 | 182 | 182 |
| UNAL:BTBC:12384 | 137 | 141 | 192 | 196 | 249 | 249 | 194 | 200 | 295 | 295 | 262 | 262 | 167 | 171 | 216 | 216 | 386 | 390 | 209 | 211 | 185 | 193 | 374 | 384 | 175 | 179 | 356 | 356 | 360 | 362 | 337 | 337 | 178 | 182 |
| UNAL:BTBC:12385 | 137 | 137 | 192 | 192 | 249 | 249 | 194 | 196 | 295 | 295 | 258 | 262 | 167 | 167 | 216 | 220 | 390 | 390 | 209 | 211 | 185 | 185 | 372 | 372 | 153 | 175 | 356 | 360 | 360 | 360 | 337 | 337 | 182 | 182 |
| UNAL:BTBC:12386 | 137 | 137 | 192 | 192 | 249 | 249 | 194 | 200 | 295 | 295 | 262 | 262 | 167 | 167 | 204 | 220 | 390 | 390 | 211 | 213 | 185 | 185 | 374 | 384 | 155 | 161 | 356 | 360 | 360 | 362 | 337 | 337 | 182 | 186 |
| UNAL:BTBC:12387 | 137 | 141 | 176 | 196 | 245 | 249 | 194 | 194 | 295 | 295 | 254 | 262 | 167 | 171 | 220 | 220 | 386 | 386 | 209 | 211 | 185 | 193 | 372 | 374 | 153 | 171 | 356 | 356 | 362 | 362 | 337 | 337 | 182 | 186 |
| UNAL:BTBC:12388 | 137 | 137 | 196 | 196 | 249 | 249 | 194 | 202 | 295 | 295 | 254 | 262 | 167 | 167 | 216 | 216 | 378 | 386 | 211 | 211 | 193 | 193 | 384 | 384 | 179 | 179 | 356 | 356 | 360 | 362 | 337 | 337 | 178 | 178 |
