# Supplementary material for: First genetic evaluation of a wild population of Crocodylus intermedius: New insights for the recovery of a Critically Endangered species
Source: PLoS One. 2024 Oct 3;19(10):e0311412. doi: 10.1371/journal.pone.0311412 (PMC11449319; doi:10.1371/journal.pone.0311412)
Supplement: S6 Table — Previous C. intermedius evaluations for ex-situ populations are indicated with an asterisk. N sample size; HO observed heterozygosity; HE expected heterozygosity; AOb observed allelic diversity; AR allelic richness. IUCN categories are NE Not Evaluated; LC Least Concerned; VU Vulnerable; CR Critically Endangered. (DOCX) [file pone.0311412.s006.docx]

| **Taxon** | **IUCN category** | **Region** | **Microsatellite nDNA** | | | | | | **Reference** |
| --- | --- | --- | --- | --- | --- | --- | --- | --- | --- |
|  |  |  | *N* | Loci | *H_O_* | *H_E_* | ***A_Ob_*** | ***A_R_*** |  |
| *C. acutus* | VU | Black River Lower Morass, Jamaica | 17 | 11 | 0.455 | 0.547 | 7.273 | – | Rossi Lafferriere and colleagues [1] |
| *C. acutus* | VU | Everglades National Park, United States | 35 | 11 | 0.562 | 0.629 | 6.091 | – | Rossi Lafferriere and colleagues [1] |
| *C. acutus* | VU | Portland Bight Protected Area, Jamaica | 82 | 11 | 0.455 | 0.545 | 3.909 | – | Rossi Lafferriere and colleagues [1] |
| *C. acutus* | VU | Turneffe Atoll, Belize | 31 | 11 | 0.497 | 0.503 | 6.364 | – | Rossi Lafferriere and colleagues [1] |
| *C. acutus* | VU | Wildlife Refuge Monte Cabaniguan, Cuba | 60 | 11 | 0.494 | 0.534 | 7.000 | – | Rossi Lafferriere and colleagues [1] |
| *C. acutus* | VU | Zapata Swamp National Park, Cuba | 14 | 9 | 0.620 | 0.640 | 5.100 | 5.100 | Milián-García and colleagues [2]; Rossi Lafferriere and colleagues [1] |
| *C. intermedius* | CR | Colombian Orinoquía, Colombia | 18 | 15 | 0.499 | 0.611 | 4.667 | 3.857 | Castillo-Rodríguez and colleagues [3] |
| *C. intermedius* | CR | Eastern Meta Basin, Colombia | 7 | 13 | 0.571 | 0.604 | 3.385 | 2.933 | Castillo-Rodríguez and colleagues [3] |
| *C. intermedius* | CR | EBTRF Alive population, Colombia* | 64 | 16 | 0.617 | 0.574 | 4.000 | 3.369 | Saldarriaga-Gómez and colleagues [4] |
| *C. intermedius* | CR | EBTRF F0, Colombia* | 37 | 16 | 0.573 | 0.587 | 4.190 | 3.852 | Saldarriaga-Gómez and colleagues [4] |
| *C. intermedius* | CR | El Frío Biological Station, Venezuela* | 20 | 17 | 0.524 | 0.544 | 5.294 | – | Rossi Lafferriere and colleagues [5] |
| *C. intermedius* | CR | Guaviare Basin, Colombia | 3 | 9 | 0.444 | 0.556 | 2.444 | 2.444 | Castillo-Rodríguez and colleagues [3] |
| ***C. intermedius*** | **CR** | **Lipa-Ele-Cravo Norte River System, Eastern Meta Basin, Colombia** | **38** | **14** | **0.592** | **0.573** | **3.857** | **3.190** | **This research** |
| *C. intermedius* | CR | Western Meta and Vichada Basins, Colombia | 8 | 15 | 0.581 | 0.626 | 3.933 | 3.858 | Castillo-Rodríguez and colleagues [3] |
| *C. mindorensis* | CR | Isabela, Philippines | 84 | 11 | 0.408 | 0.423 | 3.900 | 2.159 | Hinlo and colleagues [6] |
| *C. mindorensis* | CR | Liguasan, Philippines | 14 | 11 | 0.457 | 0.446 | 3.700 | 2.317 | Hinlo and colleagues [6] |
| *C. moreletii* | LC | Gold Button Lagoon, Belize | 9 | 42 | 0.460 | 0.410 | 3.556 | – | Dever and colleagues [7] |
| *C. moreletii* | LC | Habanero, Belize | 11 | 9 | 0.350 | 0.300 | 2.111 | – | Dever and colleagues [7]; Ray and colleagues [8] |
| *C. moreletii* | LC | New River and Gold Button Lagoons, Belize | 52 | 5 | 0.579 | 0.552 | 4.600 | – | Mcvay and colleagues [9] |
| *C. niloticus* | LC | Kenya | 17 | 12 | 0.520 | 0.420 | 3.250 | 2.400 | Hekkala and colleagues [10] |
| *C. niloticus* | LC | KwaZulu-Natal, South Africa | 10 | 11 | 0.612 | 0.745 | 4.318 | 4.919 | van Asch and colleagues [11] |
| *C. niloticus* | LC | Limpopo River Basin, South Africa | 13 | 11 | 0.480 | 0.480 | 3.450 | 2.320 | Hekkala and colleagues [10] |
| *C. niloticus* | LC | Limpopo River, South Africa | 12 | 11 | 0.717 | 0.634 | 3.818 | 3.737 | van Asch and colleagues [11] |
| *C. niloticus* | LC | Lower Kunene River, Namibia | 12 | 11 | 0.495 | 0.583 | 4.182 | 3.307 | van Asch and colleagues [11] |
| *C. niloticus* | LC | Lower Shire River, Malawi | 52 | 11 | 0.621 | 0.674 | 6.909 | 5.529 | van Asch and colleagues [11] |
| *C. niloticus* | LC | Northern Madagascar | 15 | 8 | 0.430 | 0.470 | 3.250 | 2.190 | Hekkala and colleagues [10] |
| *C. niloticus* | LC | Northwest Madagascar | 11 | 12 | 0.490 | 0.440 | 2.580 | 2.490 | Hekkala and colleagues [10] |
| *C. niloticus* | LC | Okavango River, Botswana and Namibia | 62 | 11 | 0.596 | 0.610 | 5.182 | 3.650 | van Asch and colleagues [11] |
| *C. niloticus* | LC | Okavango River, Botswana and Namibia | 142-153 | 7 | 0.510 | 0.720 | 6.700 | – | Bishop and colleagues [12] |
| *C. niloticus* | LC | Southeast Madagascar | 13 | 10 | 0.470 | 0.410 | 2.700 | 2.180 | Hekkala and colleagues [10] |
| *C. niloticus* | LC | Tanzania | 12 | 6 | 0.480 | 0.610 | 3.000 | 2.780 | Hekkala and colleagues [10] |
| *C. niloticus* | LC | West Africa | 6 | 10 | 0.400 | 0.250 | 1.720 | - | Hekkala and colleagues [10] |
| *C. niloticus* | LC | Zimbabwe | 11 | 12 | 0.570 | 0.540 | 3.330 | 2.450 | Hekkala and colleagues [10] |
| *C. palustris* | VU | Sarbaz-Bahukalat basin, Iran | 10 | 12 | 0.350 | 0.430 | 2.750 | – | Campos and colleagues [13] |
| *C. porosus* | LC | Australia | 5 | 6 | 0.633 | 0.622 | 3.830 | – | Russello and colleagues [14] |
| *C. porosus* | LC | New Britain, Papua New Guinea | 21 | 6 | 0.444 | 0.536 | 4.330 | – | Russello and colleagues [14] |
| *C. porosus* | LC | North Solomons Province, Solomon Islands | 12 | 6 | 0.458 | 0.530 | 3.170 | – | Russello and colleagues [14] |
| *C. porosus* | LC | Northeeast Papua New Guinea | 32 | 6 | 0.551 | 0.569 | 4.330 | – | Russello and colleagues [14] |
| *C. porosus* | LC | Northewest Papua New Guinea | 7 | 6 | 0.371 | 0.456 | 3.670 | – | Russello and colleagues [14] |
| *C. porosus* | LC | Palau | 39 | 6 | 0.570 | 0.575 | 4.330 | – | Russello and colleagues [14] |
| *C. porosus* | LC | Southern Papua New Guinea | 31 | 6 | 0.483 | 0.529 | 5.170 | – | Russello and colleagues [14] |
| *C. porosus* | LC | Sulawesi | 11 | 6 | 0.561 | 0.561 | 3.170 | – | Russello and colleagues [14] |
| *C. porosus* | LC | Sunda Shelf | 19 | 6 | 0.490 | 0.600 | 4.330 | – | Russello and colleagues [14] |
| *C. rhombifer* | CR | Zapata Swamp National Park, Cuba | 27 | 9 | 0.490 | 0.540 | 4.100 | 3.600 | Milián-García and colleagues [2] |
| *C. suchus* | NE | Gabbou, Mauritania | 8 | 12 | 0.470 | 0.560 | 3.330 | 2.370 | Velo-Antón and colleagues [15] |
| *C. suchus* | NE | Gorgod el Abiod, Mauritania | 6 | 12 | 0.540 | 0.610 | 3.580 | 2.590 | Velo-Antón and colleagues [15] |
| *C. suchus* | NE | Gorgol el Akhdar-Garfa, Mauritania | 4 | 12 | 0.620 | 0.740 | 3.580 | 2.870 | Velo-Antón and colleagues [15] |
| *C. suchus* | NE | Karakoro-Kolimbiné, Mauritania | 16 | 12 | 0.540 | 0.610 | 4.000 | 2.460 | Velo-Antón and colleagues [15] |

**References**

1. Rossi Lafferriere NA, Menchaca-Rodriguez A, Antelo R, Wilson B, McLaren K, Mazzotti F, et al. High levels of population genetic differentiation in the American crocodile (*Crocodylus acutus*). PLoS One. 2020;15: e0235288. doi:10.1371/journal.pone.0235288

2. Milián-García Y, Ramos-Targarona R, Pérez-Fleitas E, Sosa-Rodríguez G, Guerra-Manchena L, Alonso-Tabet M, et al. Genetic evidence of hybridization between the critically endangered Cuban crocodile and the American crocodile: implications for population history and in situ/ex situ conservation. Heredity (Edinb). 2015;114: 272–280. doi:10.1038/hdy.2014.96

3. Castillo-Rodríguez N, Saldarriaga-Gómez AM, Antelo R, Vargas-Ramírez M. Population genetic structure in the critically endangered *Crocodylus intermedius* (Crocodilia: Crocodylidae): a shift in perspective for conservation actions in Colombia. Biol J Linn Soc. 2024; 1–15. doi:10.1093/biolinnean/blad174

4. Saldarriaga-Gómez AM, Ardila-Robayo MC, Medem F, Vargas-Ramírez M. Hope is the last thing lost: Colombian captive-bred population of the critically endangered Orinoco crocodile (*Crocodylus intermedius*) is a genetic reservoir that could help to save the species from extinction. Nat Conserv. 2023;103: 85–103. doi:10.3897/natureconservation.53.104000

5. Rossi Lafferriere NA, Antelo R, Alda F, Martensson D, Hailer F, Castroviejo-Fisher S, et al. Multiple paternity in a reintroduced population of the orinoco crocodile (*Crocodylus intermedius*) at the El frío biological station, Venezuela. PLoS One. 2016;11: e0235288. doi:10.1371/journal.pone.0150245

6. Hinlo MRP, Tabora JAG, Bailey CA, Trewick S, Rebong G, van Weerd M, et al. Population genetics implications for the conservation of the Philippine Crocodile *Crocodylus mindorensis* Schmidt, 1935 (Crocodylia: Crocodylidae). J Threat Taxa. 2014;6: 5513–5533. doi:10.11609/jott.o3384.5513-33

7. Dever JA, Strauss RE, Rainwater T, Densmore LD. Genetic Diversity, Population Subdivision, and Gene Flow in Morelet’s Crocodile (*Crocodylus moreletii*) from Belize, Central America. 2002. doi:10.1643/0045-8511(2002)002

8. Ray DA, Dever JA, Platt SG, Rainwater TR, Finger AG, McMurry ST, et al. Low levels of nucleotide diversity in Crocodylus moreletii and evidence of hybridization with *C. acutus*. Conserv Genet. 2004;5: 449–462. doi:10.1023/B:COGE.0000041024.96928.fe

9. Mcvay JD, Rodriguez D, Rainwater TR, Dever JA, Platt SG, Mcmurry ST, et al. Evidence of multiple paternity in Morelet’s Crocodile (*Crocodylus moreletii*) in Belize, CA, inferred from microsatellite markers. J Exp Zool Part A Ecol Genet Physiol. 2008;309: 643–648. doi:10.1002/jez.500

10. Hekkala ER, Amato G, DeSalle R, Blum MJ. Molecular assessment of population differentiation and individual assignment potential of Nile crocodile (*Crocodylus niloticus*) populations. Conserv Genet. 2010;11: 1435–1443. doi:10.1007/s10592-009-9970-5

11. van Asch B, Versfeld WF, Hull KL, Leslie AJ, Matheus TI, Beytell PC, et al. Phylogeography, genetic diversity, and population structure of Nile crocodile populations at the fringes of the southern African distribution. PLoS One. 2019;14: e0226505. doi:10.1371/journal.pone.0226505

12. Bishop JM, Leslie AJ, Bourquin SL, O’Ryan C. Reduced effective population size in an overexploited population of the Nile crocodile (*Crocodylus niloticus*). Biol Conserv. 2009;142: 2335–2341. doi:https://doi.org/10.1016/j.biocon.2009.05.016

13. Campos JC, Mobaraki A, Abtin E, Godinho R, Brito JC. Preliminary assessment of genetic diversity and population connectivity of the Mugger Crocodile in Iran. Amphib Reptil. 2018;39: 126–131. doi:10.1163/15685381-16000173

14. Russello MA, Brazaitis P, Gratten J, Watkins-Colwell GJ, Caccone A. Molecular assessment of the genetic integrity, distinctiveness and phylogeographic context of the Saltwater crocodile (*Crocodylus porosus*) on Palau. Conserv Genet. 2007;8: 777–787. doi:10.1007/s10592-006-9225-7

15. Velo-Antón G, Godinho R, Campos JC, Brito JC. Should i stay or should i go? Dispersal and population structure in small, isolated desert populations of west african crocodiles. PLoS One. 2014;9. doi:10.1371/journal.pone.0094626
