# Supplementary figures and images for: First genetic evaluation of a wild population of Crocodylus intermedius: New insights for the recovery of a Critically Endangered species
Source: PLoS One. 2024 Oct 3;19(10):e0311412. doi: 10.1371/journal.pone.0311412 (PMC11449319; doi:10.1371/journal.pone.0311412)

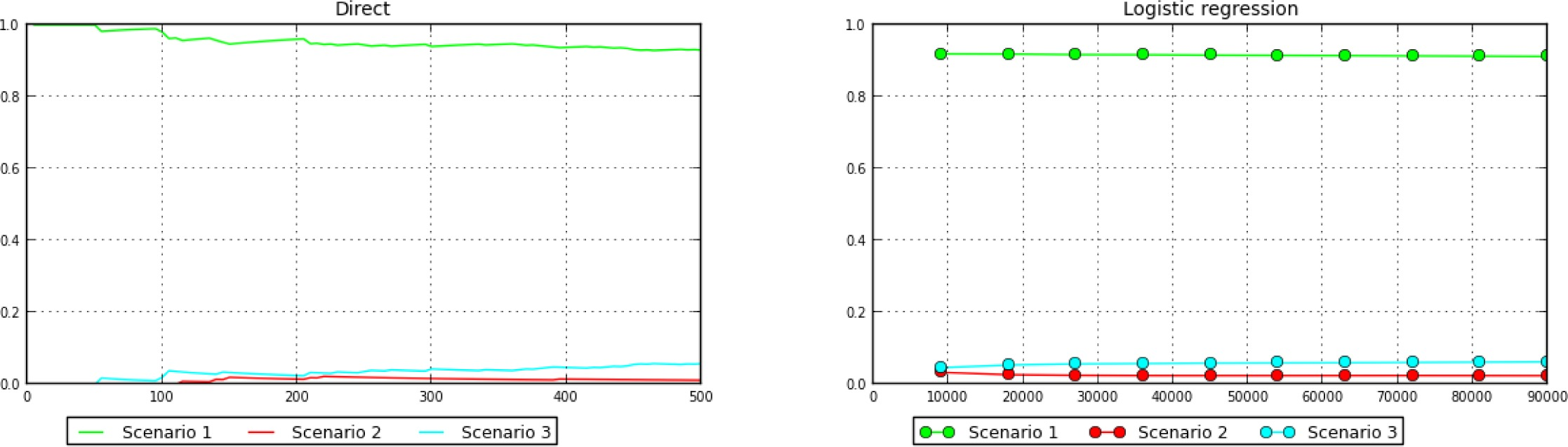

Supplement: S1 Fig — Note the strong support for Scenario 1; population decline. For the parameter settings in each scenario, refer to Materials and Methods and Fig 2. (TIF) [file pone.0311412.s007.tif]

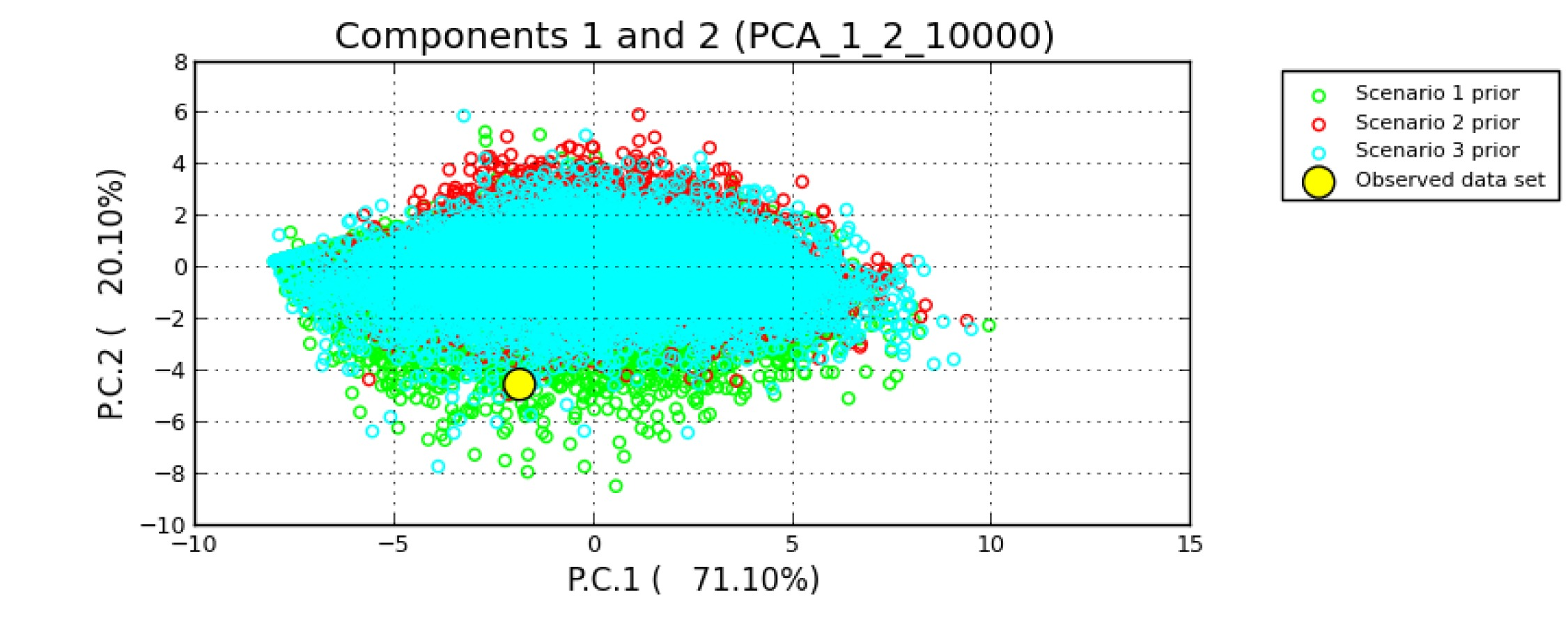

Supplement: S2 Fig — It confirms that the model fits well, as the genetic data fall within the range of the simulated results. (TIF) [file pone.0311412.s008.tif]

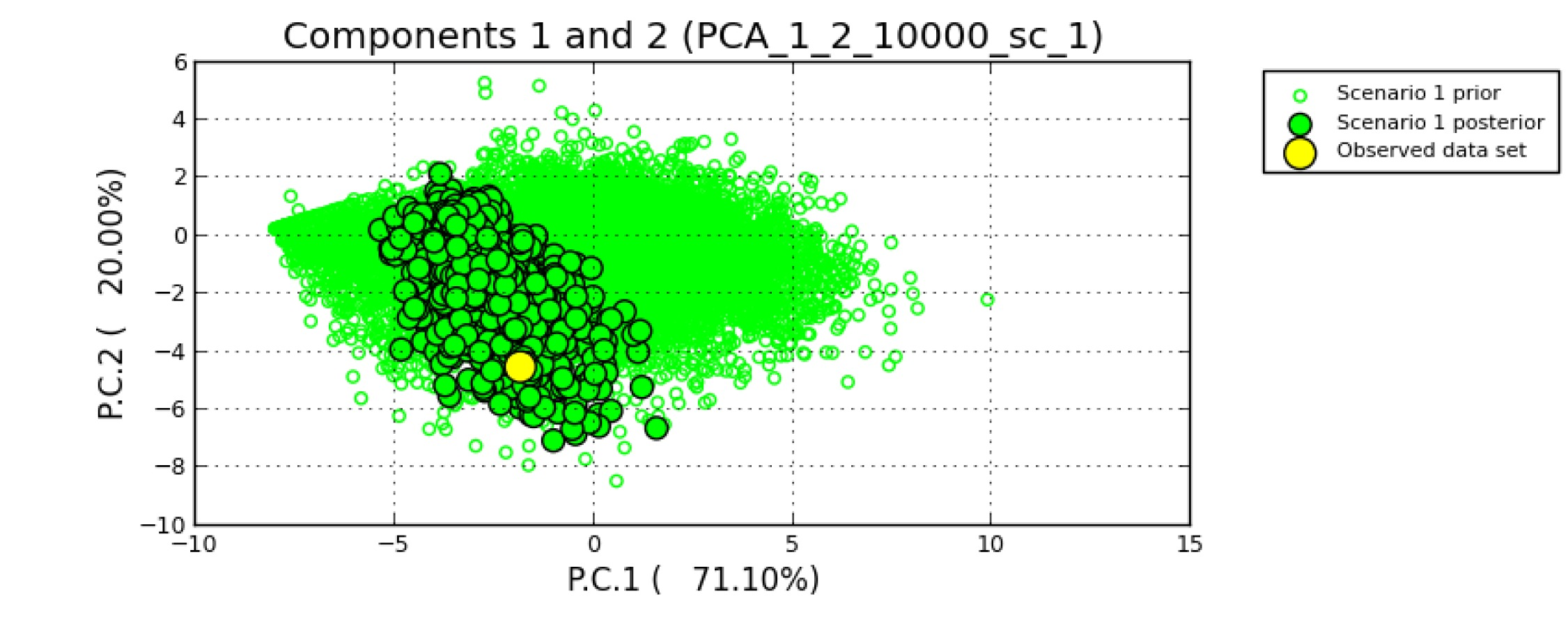

Supplement: S3 Fig — It shows the observed data set summary statistics value and proportion of datasets (simulated from the posterior) that have a value lower than the observed dataset. (TIF) [file pone.0311412.s009.tif]

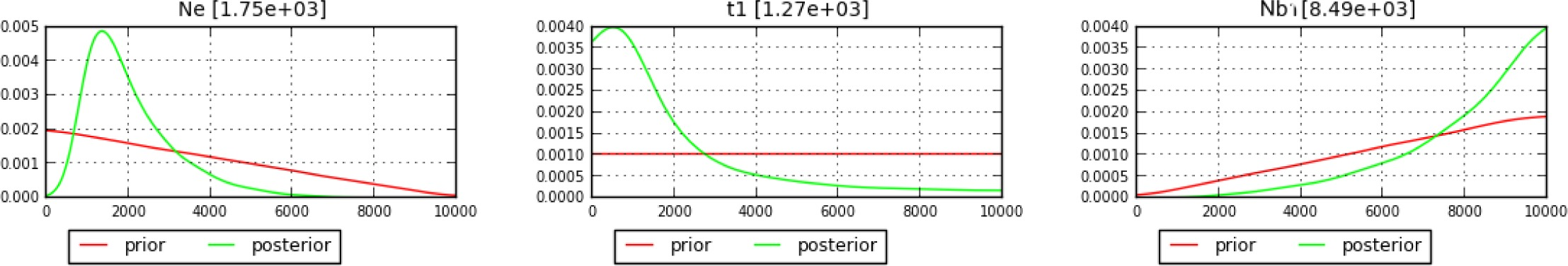

Supplement: S4 Fig — Times are not scaled. (TIF) [file pone.0311412.s010.tif]
